# Silicon allotropes by large-volume high-pressure techniques: crystal growth mechanisms, phase diagrams and hexagonal nanostructured Si-6H by *in situ* X-ray diffraction and computational methods

**DOI:** 10.1107/S2052520626004026

**Published:** 2026-06-01

**Authors:** Alexandre Courac, Yann Le Godec

**Affiliations:** a Institut de Minéralogie, de Physique des Matériaux et de Cosmochimie (IMPMC) Sorbonne Université UMR CNRS 7590 Muséum National d'Histoire Naturelle IRD UMR 206 Paris 75005 France; b Institut Universitaire de France (IUF) Paris 75005 France; Siberian Branch of Russian Academy of Science, Russian Federation

**Keywords:** *in situ*X-ray diffraction, hexagonal silicon, high pressure and high temperature, phase transformations, large-volume high-pressure devices

## Abstract

Metastable allotropes of silicon recovered from high-pressure conditions exhibit a wide range of crystal structures, physical properties and transformation pathways that remain only partially understood despite decades of study. This article combines original crystallographic observations with a critical review of phase transformations, nucleation mechanisms and crystal growth processes in elemental Si and Na–Si systems synthesized under high-pressure, high-temperature conditions. Using *in situ* diffraction data, structural characterization and computational approaches, we analyze how symmetry breaking, lattice instabilities and kinetic constraints govern the formation of dense polymorphs (Si-II, Si-III, Si-XI) and open-framework structures, including clathrate and channel phases. Particular attention is given to the role of large-volume synthesis and chemically assisted growth routes in controlling phase selection, defect formation and recoverability. The evolution of hexagonal polytypes, including nanostructured 6H silicon, is discussed in terms of stacking modifications driven by stress release and thermal treatment. By integrating crystallographic relations, thermodynamic considerations and growth kinetics, this work identifies phase-transformation mechanisms as the key factor linking structure, synthesis conditions and functional properties of silicon allotropes. The results provide a unified framework for understanding crystal growth at high pressure and offer guidance for the controlled synthesis of advanced silicon materials.

## Abstract 2

*In situ* X-ray diffraction is used for exploring Si crystallography and for studying phase-transformation mechanisms in elemental Si and Na–Si systems. We present original results of hexagonal silicon, Si-6H and a critical analysis of the past-decade discoveries of silicon Si-4H and Si_24_ using large-volume high-pressure techniques.

## 1. Introduction and scope

Fundamental interest drove the initial high-pressure (HP) study of silicon (common Si with diamond structure known as Si-I) (Minomura & Drickamer, 1962 ▸), which led to the early discovery of so-called Kasper phases Si-III and Si-IV (Wentorf & Kasper, 1963 ▸). These phases are metastable under ambient conditions and, presumably, have no stability domain at any *p–T*,*i.e.* they are intrinsically metastable. The majority of available HP techniques employed for Si synthesis and studies are given in Table 1 ▸. Direct phase transformations and chemically assisted growth are currently two major routes toward obtaining large (over mm-sized) samples (Le Godec & Courac, 2021 ▸), using principally so-called large-volume HP techniques (LVP or large-volume presses), which are central to this review. The results of other techniques will be mentioned only when relevant to this primary scope.

**Figure 1:**
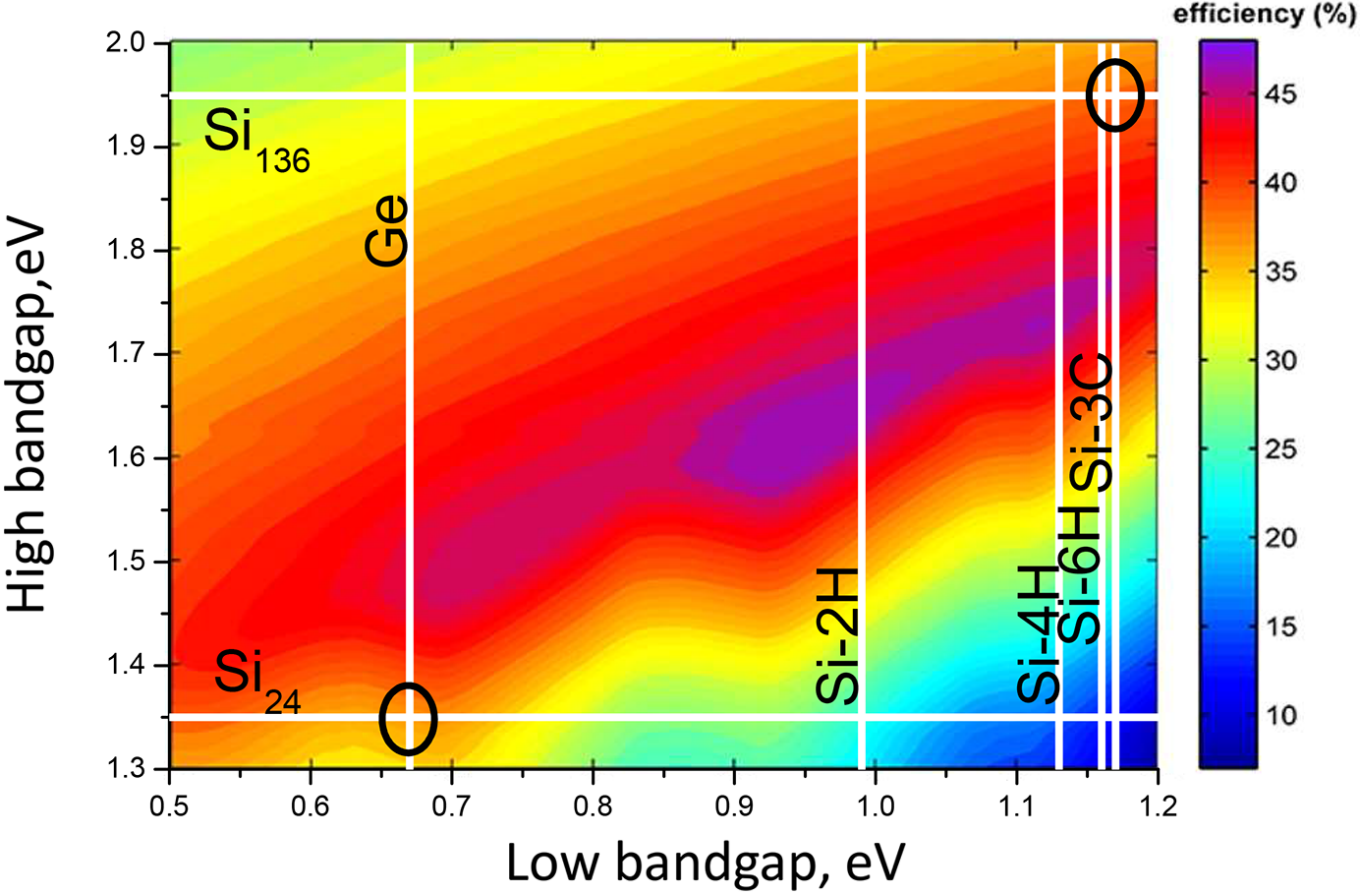
The maximal theoretical efficiency of tandem cells (Bremner *et al.*, 2008 ▸). Si forms and Ge are shown as white lines. Vertical and horizontal lines correspond to low- and high-bandgap semiconductors of the cell, respectively. Ovals encircle mentioned in the text examples of Ge+Si_24_ and Si-3C+Si_136_.

Si is widely used in photovoltaics. However, due to incomplete absorption, thermalization, thermodynamic loss and radiative recombination, the maximal efficiency is ∼33% (so-called Shockley–Queisser efficiency) (Shockley & Queisser, 1961 ▸). Typically, Si cells have ∼20% efficiency. Si is cheap, non-toxic, easily purified and doped. No stoichiometry problem arises, unlike in many compounds. To increase the efficiency, tandem cells are possible (Bremner *et al.*, 2008 ▸). As an example, Fig. 1 ▸ shows that Ge+Si_24_ or Si-3C+Si_136_ tandem cells are promising couples with efficiency of up to 40%.

**Figure 2:**
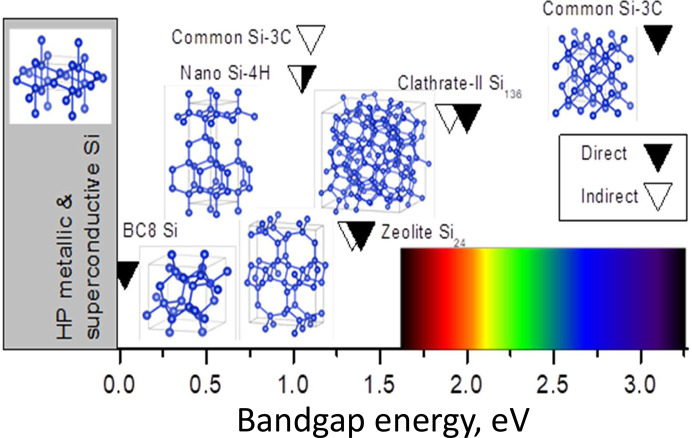
Structure–bandgap map of Si phases. Si semiconductors known as metastable allotropes that can be produced in one- or two-step synthesis in Si or Na–Si systems. At least one step includes HP.

Solid-state design of novel silicon forms can be performed either (i) by using *in situ* techniques to explore experimentally accessible pressure–temperature composition space, as well as strain, which can be either intentionally applied, or intrinsic to phase transformations involving volume changes, or (ii) by structural generation and evolution in the framework available algorithms combining *ab initio* calculations, meta­dynamics, machine learning, and structural optimization criteria in terms of the energetic stability or desired property, *etc*. Selection of the best Si candidates – remarkably less numerous in experimental studies as compared to theoretical ones – is typically performed using criteria such as optically allowed direct bandgap of ∼1–2 eV corresponding to high Shockley–Queisser efficiency (Shockley & Queisser, 1961 ▸). Close direct and indirect bandgaps are also desired. Crystallographic match between both structures can play an important role for the creation of efficient contact between two semiconductors, and can be linked to the possibility of mutual phase nucleation and, thus related to kinetics and mechanism. The typical solutions for Si bandgap engineering consist of adapting nanostructuring, dimensionality, and the crystal structure. For example, nanostructuring can remarkably increase the bandgap that can be experimentally observed by enhanced emission with a blue shift of the photoluminescence (Delerue *et al.*, 1998 ▸). Confinement in two directions, in the case of Si nanowires, leads to the indirect-to direct bandgap transition (Jensen *et al.*, 2016 ▸). The most powerful tool for bandgap engineering – but also the most complicated experimentally – is crystal structure change. This last method enables a large variety of bandgaps to be covered. Experimentally (Fig. 2 ▸), silicon crystalline forms that can be accessible at ambient conditions, show bandgaps from 30 meV for narrow-bandgap dense Si-III of cubic BC_8_ type structure (Zhang *et al.*, 2017 ▸) to ∼2 eV for open-framework clathrate Si_136_ (Gryko *et al.*, 2000 ▸). Nanosize and shape impact the phase transformation pressure (Yesudhas *et al.*, 2024 ▸; Huston *et al.*, 2021 ▸), as well as the initial crystal structure and pressure medium (Barkalov *et al.*, 2021 ▸). The possibility of directly observing new crystal species under high pressure and high temperature (HPHT) conditions through the development of *in situ* crystallography beamlines has revolutionized the field (Guignard & Crichton, 2015 ▸).

Both experimentally known and predicted structures can have higher or lower density than common Si-I. Thus, dense phases may be formed by HP synthesis, for example Si-II, Si-III, Si-V, Si-XI, Si-XII, *etc.* (Tables 2 ▸ and 3 ▸); but not all of them can be recovered after decompression as a phase-pure sample in large volume. Si-II was once reported recoverable at 100 K (Imai *et al.*, 1996 ▸), while previously reported tetragonal Si-VIII and Si-IX phases (Zhao *et al.*, 1986 ▸), never reproduced, could be misinterpretation of the limited diamond anvil cell (DAC) diffraction data. Si-III stability is questioned in the literature, and is considered as possibly being extrapolated from strain experiments (Blank & Estrin, 2013 ▸). Si-III, once recovered and heated under vacuum or inert atmosphere, transforms into hexagonal silicon Si-IV (Wentorf & Kasper, 1963 ▸), which has been recently established to be a 4H polytype (Si-IV 4H) of diamond silicon 3C (Si-I 3C) (Pandolfi *et al.*, 2018 ▸), and not 2H as was previously believed. Thus, HP phases can also serve as precursors for other dense Si forms by further transformations using other ambient-pressure techniques.

For open-framework phases with lower density, which correspond to thermodynamic stability at negative pressure, the synthetic route is quite different. In fact, first one should produce an HP phase with desired Si framework, and, in a second step, remove intercalated atoms, which is possible, for example, by evaporation or electrolysis. An analogy to hydro­thermal zeolite synthesis can be made (Cundy & Cox, 2003 ▸), while known open-framework clathrates Si_136_ (Kasper *et al.*, 1965 ▸) and Si_24_ (Kim *et al.*, 2015 ▸) and their precursors Na_24±*y*_Si_136_ (Kasper *et al.*, 1965 ▸; Yamanaka *et al.*, 2014 ▸) and Na_4_Si_24_ (Kurakevych *et al.*, 2013 ▸; Guerette *et al.*, 2018 ▸) have zeolite-type structures.

Pressure is an important factor that influences transformations in elemental Si and its forms, and fills silicon frameworks with an intercalated atom (particularly alkaline, alkali-earth, halogenides and hydrogen), and that can be subsequently removed in some cases. In this paper, we attempt (i) to assemble the available data on pressure-induced transformation in pure silicon and silicon frameworks filled with sodium at ambient and high temperatures (HT), and (ii) analyze the variety of mechanisms responsible for transformations and chemical reactions that underlay the design of advanced Si forms, particularly those that can be directly proved experimentally using X-ray diffraction (XRD).

Efficient crystal growth at HPHT requires the knowledge of phase-transformation mechanisms. The underlying mechanism of metastable allotrope synthesis—and all known HP Si materials are intrinsically metastable (Fan *et al.*, 2021 ▸)—can be partially understood by the thermodynamics of phase equilibria in Si and Na–Si system at HPHT conditions. In the case of direct phase transformations, Si-II formation is a crucial step, and in the case of Na-assisted synthesis, the clathrate formation is required. Kinetic factors play an important role in the recovery of metastable phases by direct phase transformations (Wang *et al.*, 2013 ▸), as well as in solvent-assistant synthesis (Guerette *et al.*, 2018 ▸). In this paper, five further sections are presented: *2. Structural landscape of metastable silicon*[Sec sec2], 3. *Dense silicon allotropes*[Sec sec3], 4. *Chemically assisted crystal growth in Na–Si systems*[Sec sec4], 5. *Modeling and integration*[Sec sec5], and 6. *Outlook and perspectives*[Sec sec6]. The present review is intentionally not exhaustive, rather, it is complementary to a recommended review by Haberl *et al.* (2016 ▸, and references therein). The main objectives of the present review are to formulate key questions and the hypothesis related to thermodynamic and kinetic aspects of mechanisms responsible for exotic Si phase formation in solid/solid or solid/liquid interfaces and how they can be probed with available *in situ* techniques. This article demonstrates that phase-transformation mechanisms constitute the fundamental link between crystal structure, thermodynamics, and crystal growth of silicon allotropes under HP conditions. Si-6H has been characterized using XRD as an individual microscopic bulk phase, previously suggested primarily as a crystallographic curiosity.

## 2. Structural landscape of metastable silicon

Stable and metastable crystal structures will be described in this section. Often stability is graphically represented as a negative thermodynamic potential (Gibbs or Landau) over continuous thermodynamic or structural parameters. The first type of landscape is useful for experimental comparison of known phases, while the second one is useful for theoretical conceptualization and guided choice between hypothetical structures (Wang *et al.*, 2014 ▸).

### 2.1. Metastable silicon allotropes: structures and properties

HP induces the transformation of common cubic diamond Si to tetragonal β-tin Si-II, orthorhombic Si-XI and simple hexagonal Si-V at pressures available for large-volume samples (*e.g.* below 20 GPa). Typically, at 300 K, no HP phases are recovered. Instead, body-centered cubic BC8 Si-III can be recovered in large-volume experiments, while rhombohedral R8 Si-XII can be observed occasionally, in contrast to DAC experiments (Tables 2 ▸ and 3 ▸). Si-III produced in multianvil experiments is a starting HP material for a family of hexagonal silicon (Si-IV) established to be a 4H polytype (Si-IV 4H) of diamond silicon 3C (Si-I 3C) (Pandolfi *et al.*, 2018 ▸), and even pure 6H at higher temperatures (current results). Hexagonal Si (2H) definitely exists, but requires alternative methods of synthesis, like CVD.

It is quite difficult to give exhaustive and comprehensible names to all diverse Si allotropes. Tables 2 ▸ and 3 ▸ represent the most common of them. For example, common silicon phase may be denoted as (i) Si-I, (ii) Si-3C, (iii) Si-A4 and (iv) Si-cF8. They respectively indicate (i) the order of allotrope discovery, I; (ii) the *ABC* polytype of diamond structure in hexagonal setting, 3C; (iii) the general classification of crystal structures where A4 stands for diamond, so-called *Strukturbericht* symbols; and, finally, (iv) Pearson symbol indicating symmetry (c for cubic), unit-cell type (F for face-centred) and number of atoms per unit cell (8).

Open framework structures are generally obtained via chemical routes, either by soft chemistry or by HPHT synthesis of the precursor compound, which are generally HP phases. We typically follow the common notation indicating the number of atoms per unit cell, such as Si_136_ for cubic clathrate of type II or Si_24_ for orthorhombic zeolite-like phases. Some residual Na atoms were suggested in crystallographic studies (Cros & Pouchard, 2009 ▸).

### 2.2. Experimental platforms for large-volume HP silicon research

#### 2.2.1. Pressure, temperature, and hardness issues

Under HP, silicon, either crystalline or amorphous, undergoes multiple transformations that can be easily revealed by *in situ* electrical (Minomura & Drickamer, 1962 ▸), Raman (Weinstein & Piermarini, 1975 ▸), visible and IR absorption (Welber *et al.*, 1975 ▸) and/or X-ray diffraction (Olijnyk *et al.*, 1984 ▸) measurements. *Ex situ* characterizations on recoverable microscopic mm-sized samples of Si-III and products of its transformations Si-IV have been recently performed by numerous techniques, including ^29^Si NMR (Kurakevych *et al.*, 2016 ▸; Pandolfi *et al.*, 2018 ▸), IR absorption and reflection (Kurakevych *et al.*, 2016 ▸; Zhang *et al.*, 2017 ▸), low-temperature heat capacity (Zhang *et al.*, 2017 ▸), *etc*. For post-indentation surface investigations, *ex situ* Raman spectroscopy together with combined transmission electron microscopy (TEM) and electron diffraction analyses were primarily employed (Haberl *et al.*, 2016 ▸). *In situ* HT indentation has been recently proposed as a promising method of covering the Si single crystal surface with hexagonal Si mosaic nanostructures (Sasidharan Nisha *et al.*, 2025 ▸).

Temperature can also be applied to various forms of Si. When it is done at HP, one can talk about direct HPHT synthesis (Demishev *et al.*, 1996 ▸; Kurakevych *et al.*, 2016 ▸; Pandolfi *et al.*, 2018 ▸). In addition, when it is applied to recovered HP forms of Si, we deal with coupling of HP with conventional synthetic methods, or two-step synthesis (Wentorf & Kasper, 1963 ▸; Kurakevych *et al.*, 2016 ▸; Pandolfi *et al.*, 2018 ▸).

**Figure 3:**
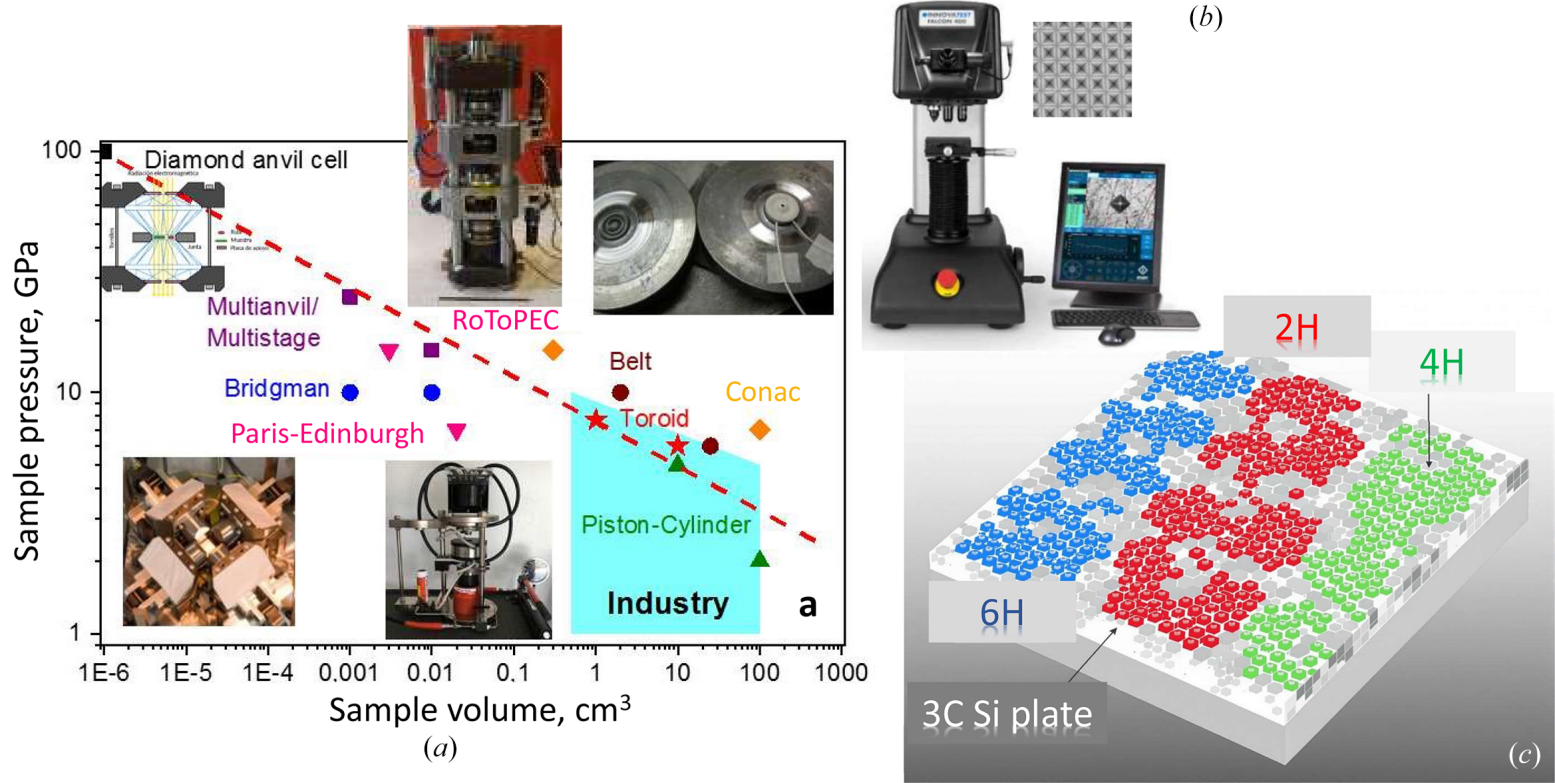
(*a*) Achievable pressure as a function of reaction volume for various HPHT apparatuses (inserts: schematic diamond anvil cell, multianvil at ESRF (France), piston-cylinder at IMPMC/France, toroid at LSPM (France). (*b*) Microhardness (Vickers & Knoop) tester at IMPMC, Sorbonne University (France). (*c*) Mosaic areas of hexagonal polytypes can be identified using electronic diffraction and typical atomic arrangements on indented 111 single-crystal Si surface (Sasidharan Nisha *et al.*, 2025 ▸).

HP can be generated in a large reaction volume by uni- or three-axial HP apparatuses [Fig. 3 ▸(*a*)] or over a large surface by indentation with a diamond indenter of various shapes [Vickers and Knoop, see Fig. 3 ▸(*b*)]. Most of these techniques are compatible with *in situ* physical measurements (Table 1 ▸). Pressure (force by unit area in a given direction) is often evaluated by the atomic volume change of a phase at given temperature using known equation of state *V*(*p*,*T*) and experimental XRD data on crystallographic density. The hardness value has the same dimensions as pressure (and often expressed in GPa for very hard materials), and is evaluated by surface resistance to diamond indentor penetration, *i.e*. by a ratio between applied force and indentation area. An intrinsic link between hardness and bandgap has been previously suggested for Si (Gilman, 1993 ▸) and other semiconductors, which, however, is questionable in light of the recent discovery of the narrow bandgap of Si-III, which exhibits relatively high hardness (above 12 GPa). The mechanism of hardness and transformations in Si are thus to be reconsidered.

#### 2.2.2. Large-volume presses (multianvil, Paris–Edinburgh) versus DAC and indentation

In the present paper, mainly large-volume synthesis will be discussed (Table 2 ▸). A recent review (Le Godec & Courac, 2021 ▸) contains extended and clear methodological sections, particularly the descriptions of HP apparatuses installed at synchrotron beamlines. Good reviews of diamond-anvil cell and indentation experiments are also available in a book chapter (Kiran, Haberl *et al.*, 2015 ▸) and paper (Haberl *et al.*, 2015 ▸). Furthermore, *in situ* experiments that combine HP with severe plastic deformation (most notably HP torsion) performed in a rotational diamond anvil cell (RDAC) (Blank *et al.*, 2019 ▸) for sub-micrometric samples, or in a RoToPEc device (Philippe *et al.*, 2016 ▸) for micrometric samples, have recently garnered significant attention within the HP materials community due to their relevance for materials processing, mechanochemistry, and geophysical applications (Levitas, 2019 ▸). Remarkably, recent studies have demonstrated that applying HP torsion to silicon enables the formation of nanostructured metastable Si-III and even Si-XII phases using an RDAC setup. These findings are particularly promising and motivate ongoing efforts to reproduce and extend these results using the larger-volume RoToPEc apparatus.

Amorphous silicon, which has close-range order similar to the Si-I crystalline form and is denser than its crystalline counterpart, is also a promising starting material that will not be considered here. It can be used for large-scale materials design, like Si-I, and has also been studied under HP. It shows somewhat different behavior in the terms of sequences of metastable crystalline phases and onset pressure of phase transformations (Haberl *et al.*, 2013 ▸).

Shock compression can also be used for production of exotic forms of silicon, similar to how it is done in the case of nano-diamond. Some recent advances in this field can be found in the literature (Pandolfi *et al.*, 2022 ▸; McBride *et al.*, 2019 ▸)

#### 2.2.3. Data comparison problem

At the same time, it is important to underline some details regarding accuracy, reproducibility, and limitations of such *in situ* experiments – the best available to date for studies of the mechanism of phase transformation. High elastic constants and 3D crystal framework rigidity that is limited only by phase transition, rather than by plasticity, allow the accumulation of significant stress by transforming the crystal structure (up to 5 GPa for Si-I as compared to transition pressure, and 1–2 GPa as compared to pressure medium or the starting Si-I phase for softer metallic Si-II and Si-XI). Typical reproducibility of multianvil experiment is ∼0.5 GPa (as declared) or even 1 GPa, and can be higher in reality. This is crucial for analyzing and comparison of data from different sources, especially for phase transformations between phases with pressure or temperature intervals of stability comparable to the measurement error. Even if pressure is determined precisely using an internal standard (such as Si), the irreproducibility can be caused by other factors, either well identified such as time, strains, pressure medium or unknown parameters. In this review the data analysis will be made in close relationship to the model or hypothesis to be tested (*e.g.* second- or first-order phase transition between Si allotropes) and not *per se*. Unambiguous high-time-resolution experiments under hydro­static conditions are expected to shed more light on the subject. The classic, while often ignored, annealing technique that consists of sample heating to a couple of hundred K prior to measurements, could also be useful.

Phase transformations in Si start at above 10 GPa, which also limits the hardness value to ∼10 GPa (for single crystals). The most probable hardness mechanism to break is the resistance of the Si-I surface, due to the phase transformation into the remarkably denser phase Si-II. Raman and TEM studies indirectly confirm this mechanism by observation of Si-III and Si-XII (Kiran, Haberl *et al.*, 2015 ▸). The contact electrical conductivity method during indentation at HT is also a promising *in situ* tool for systematic study of surface modification of Si forms (Kiran, Tran *et al.*, 2015 ▸).

Crystalline Si is a quite hard material (Table 3 ▸), and the structure, therefore, should be able to accumulate quite important stresses and strains prior to transition. To clearly observe the transformations, one needs a pressure medium (preferably a liquid to ensure hydro­static conditions or constant pressure all over the reaction volume). This factor enables quite a broad range of Si-I/Si-II phase coexistence in the case of a Si-I sample without a pressure medium (Kubo *et al.*, 2008 ▸), while in the case of hydro­static and quasi-hydro­static conditions (liquid and soft-solid pressure media), the transformations occur practically without a phase coexistence domain (Anzellini *et al.*, 2019 ▸).

Systematic studies are possible only with thorough analysis of hydro­staticity, temperature control, dwell time, compression time scale, and recovery conditions. We attempted to do it whenever possible, or to indicate the lack of information.

#### 2.2.4. Irreproducible phases: Si-VIII and Si-IX

Multiple phases have been claimed based only on a powder X-ray diffraction pattern, without a rigorous crystallographic analysis. Some of them have been proven later, such as γ-B (Oganov *et al.*, 2009 ▸) despite its complicated structure (Oganov *et al.*, 2011 ▸), while most of them were either refuted or simply never confirmed. Another example is the B_6_N phase (Hubert *et al.*, 1998 ▸), whose original powder XRD data remain enigmatic. The data was reproduced, and at the same time, the proposed crystal structure was refuted (Solozhenko *et al.*, 2006*a* ▸), but not resolved. The HP synthesis implies a multi-material sample environment, and therefore, possible contaminations. For example, the XRD contribution (i) of the Au capsule led to the data over interpretation of a diamond-structure B_2_O phase (Endo *et al.*, 1987 ▸), (ii) of NaCl pressure medium for NaCl-like MgC (Shul’zhenko *et al.*, 1988 ▸), or (iii) of HP NaCl allotrope for new BC_3_ species (Zinin *et al.*, 2012 ▸); the list can be continued. A list of unidentified *hkl* such as for BC_1.6_ (Zinin *et al.*, 2006 ▸) or graphite-like B_2_O (Hall & Compton, 1965 ▸) can also lead to various hypotheses, such as constant composition with crystal structure diversity or an individual phase. In the BC_*x*_ case it is difficult to recognize all the phases under chemical decomposition to boron carbides (Solozhenko *et al.*, 2009 ▸). As for B_2_O, its graphite-like powder diffraction pattern can be reproduced by a simulated mixture of phases of the B–O system, obtained at the same *p–T–x* conditions (Solozhenko *et al.*, 2006*b* ▸). Numerous examples for light elements can be found in the review by Kurakevych (2009 ▸). Typical low quality of reported data, both DAC or large volume, does not allow unambiguous attribution of all *hkl* reflections. Sometimes only intensities do not coincide, while phase composition seems correct (Hubert *et al.*, 1998 ▸). In many cases the diffraction of the capsule, the pressure gauge and/or pressure-medium materials were not checked. At the same time, no conventional refute procedure is available so far, and the majority of irreproducible phases remain a matter of private communications.

Here, we applied simulations of powder XRD patterns for the proposed sample environments in order to analyze the powder XRD reported for the claimed Si-VIII and Si-IX phases in a DAC (Zhao *et al.*, 1986 ▸). The powder XRD data simulated for a mixture of Si-III, Si-XII and Al_2_O_3_ (pressure gauge) was compared with experimental patterns (Figs. S2 and S3). Texturing of the sample and special phase segregation can explain the absence of some reflections, but are not sufficient to explain all observed patterns. Other phases that could be possible in this system (known Si allotropes, diamond) were not consistent with reported data.

#### 2.2.5. *In situ* LVP probes: XRD, electrical resistance, calorimetry

*In situ* techniques allow the transformation to be followed in real time, both qualitatively and quantitatively. Even in the case of multiphase samples, the *hkl* reflections of an individual phase appear at the same time, thus facilitating the recognition or at least attribution to an individual phase. Molar volumes of elemental Si and its compounds, as well as reaction volumes in the Na-Si system with participation of liquid phase, can be efficiently probed at HPHT conditions using the recent development of specialized synchrotron beamlines, electrical cells and HP calorimetry.

**Figure 4:**
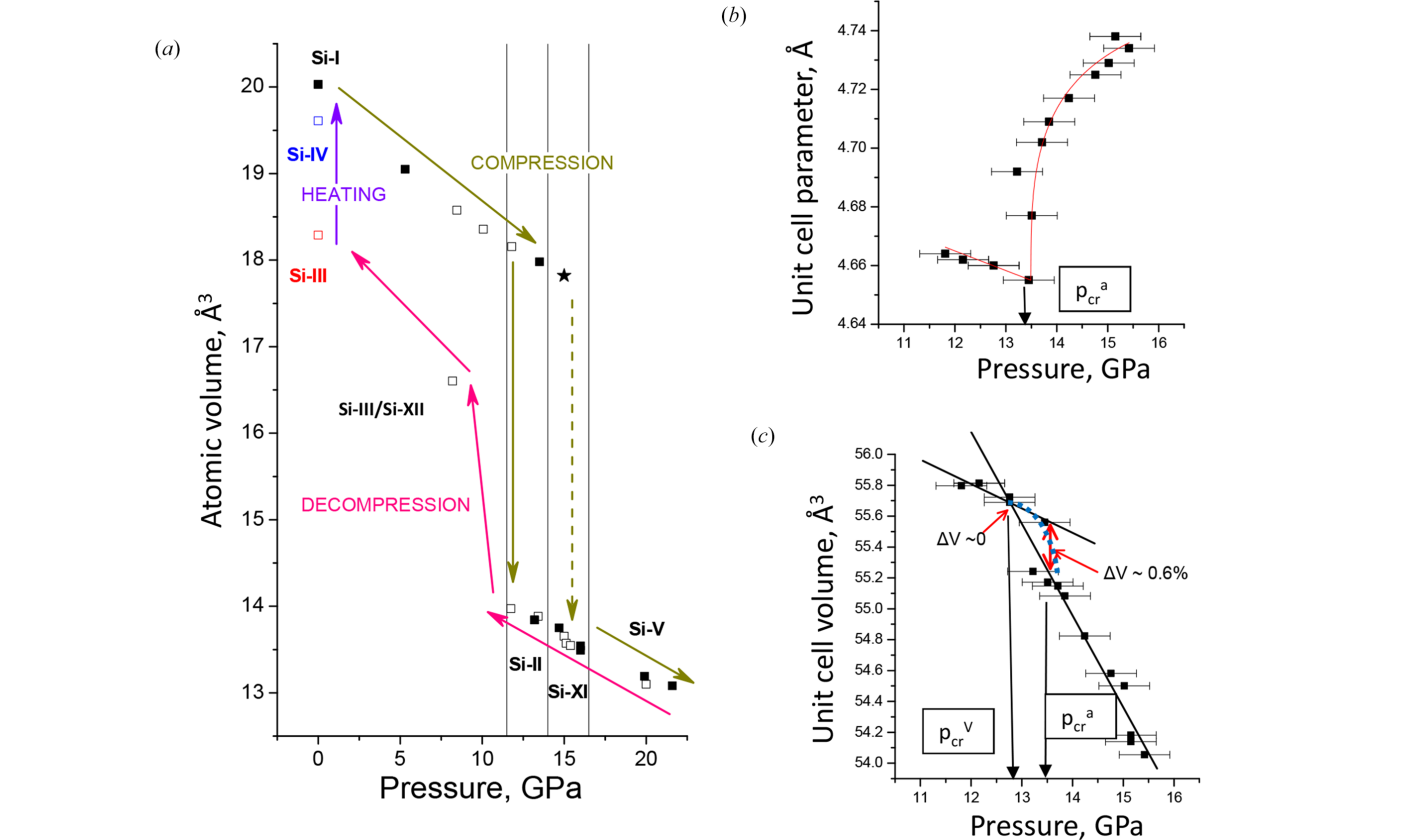
(*a*) *In situ* atomic volume of Si that shows hysteresis on compression cycle at 300 K, starting from Si-I and on the recovery of Si-III. Heating is required to get into the state with initial volume (Si-I) via hexagonal Si-IV polytypes. (*b*) Critical behavior of unit-cell parameter *a* of Si-II, present fit (red line) gives critical exponent of ∼0.31. (*c*) Low compressibility of Si-II as compared to higher compressibility of Si-XI. Ambiguity of volume change *ΔV* during transformation of Si-II to Si-XI: *ΔV* at critical point versus *ΔV* at equilibrium. Possible time-retarded kinetic domains are indicated in blue. Black lines indicate the thermodynamic expectation of EOS.

**Figure 5:**
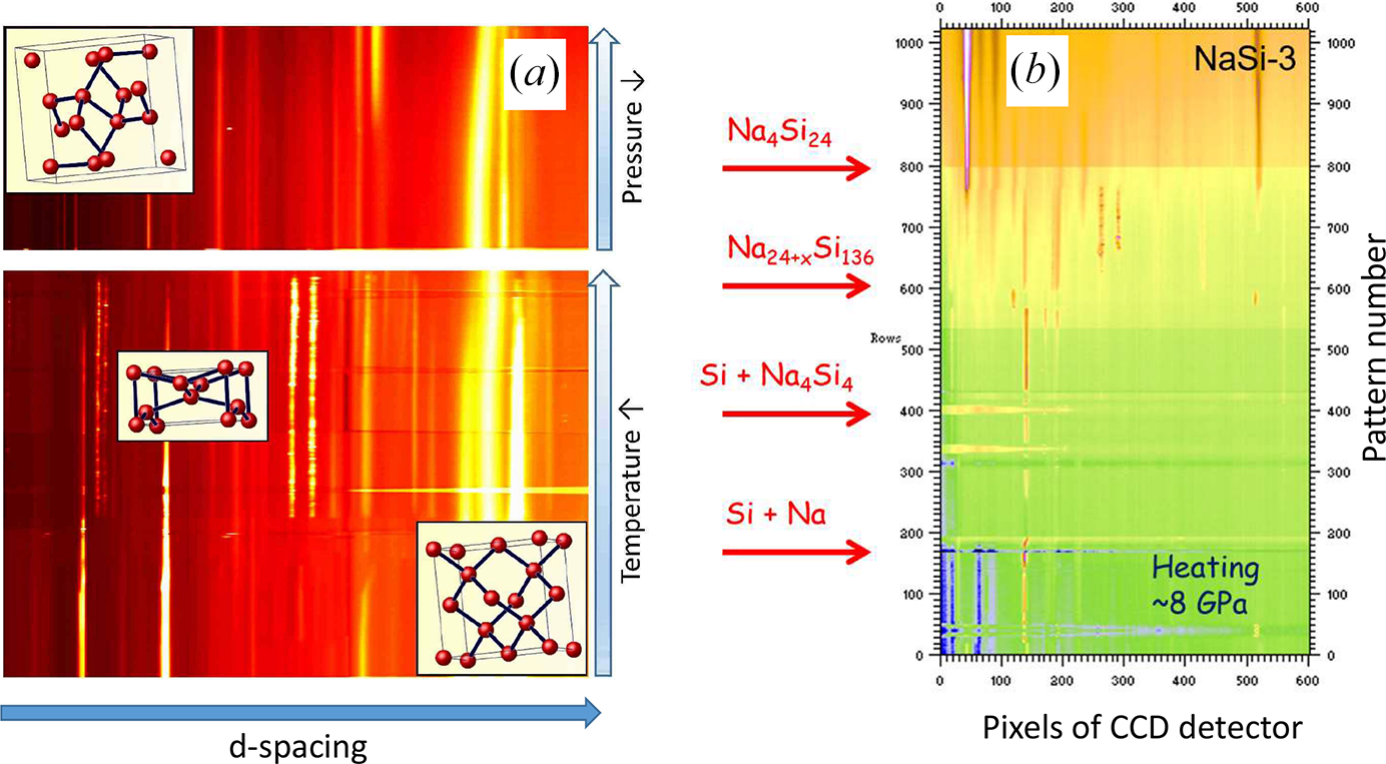
Typical *in situ*X-ray diffraction data on crystallization of Si-III with BC8 structure by direct phase transformations Si-I → Si-II → Si-III (inserts show corresponding crystal structures) (*a*); and (*b*) raw data of observation of Na_4_Si_24_ formation by the sequence of chemical reactions: Si-I (+Na) → Si-I (+ Na_4_Si_4_) → Na_30.5_Si_136_ → Na_4_Si_4_ (+ Si-II) obtained at beamline ID06 of ESRF.

Direct phase transformations in Si can be revealed by typical *in situ* data on crystallographic density (or atomic volume, see Fig. 4 ▸), while the synchrotron beamlines allow getting the high quality time-resolved data on volumes up to a couple of mm^3^ [Fig. 5 ▸(*a*)].

**Figure 6:**
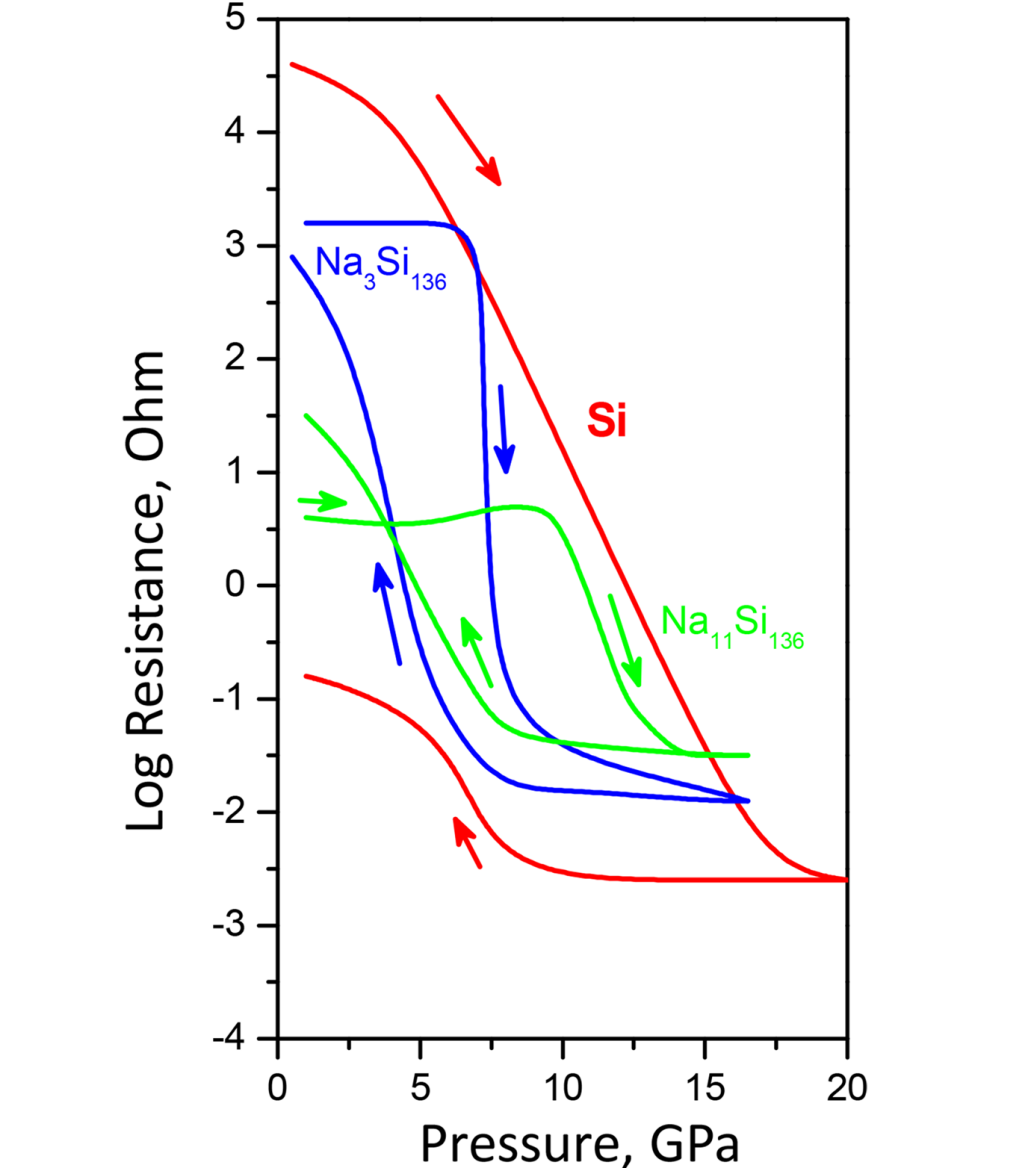
Original (Bundy, 1964 ▸; Bundy & Kasper, 1970 ▸) electrical measurement on Si and Na–Si clathrates with reversible sharp phase transitions of clathrate II, as compared with an irreversible broad transition in Si-I.

**Figure 7:**
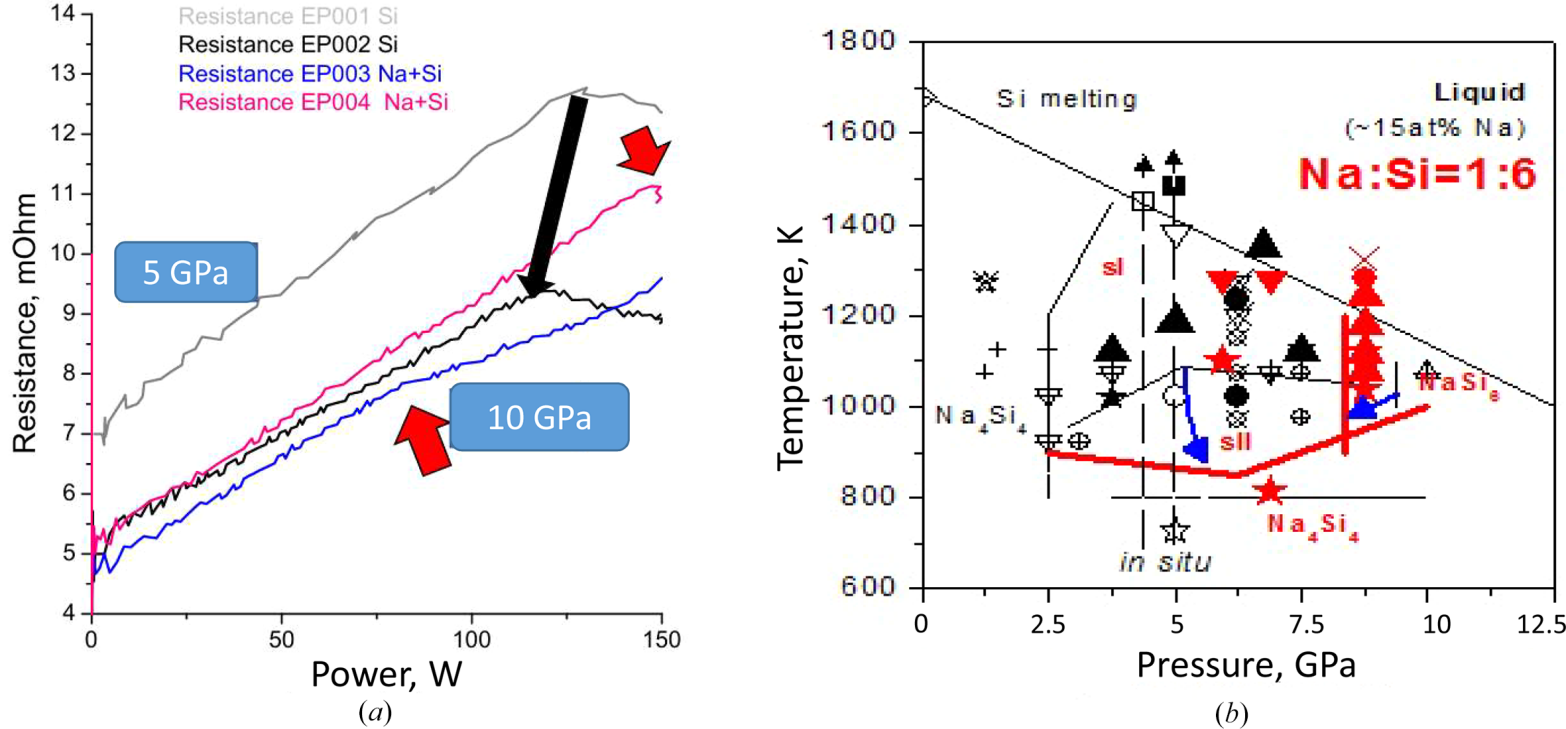
*In situ* resistance measurements. (*a*) Raw data on qualitative HPHT calorimetry. Black arrow shows pressure evolution of maximum power corresponding to melting of pure Si. Red arrows indicate the deviation from resistance–power linearity due to the formation of a metallic (clathrate) phase. (*b*) Tentative isoplethic section of a phase diagram obtained in experiments with (in black) Na:Si = 1:5.5 (Jouini *et al.*, 2016 ▸) and (in red) at Na:Si = 1:6. Blue arrows show stability domain evolution (sI, sII and Na_4_Si_24_), with Na:Si ratio from 1:5.7 (black) to 1.6 (red) .

Under pressure, sodium clathrates show phase transitions with change of electrical properties. For example, silicon clathrates Na_*x*_Si_136_ show phase transitions under compression with drop of resistance, which was observed in 1965 (Bundy & Kasper, 1970 ▸) (Fig. 6 ▸). In past decades, the electrical measurements was performed at high temperatures, up to Si melting (Courac *et al.*, 2019). The convincing results of qualitative calorimetry of Na–Si samples confined in either a metallic or graphite heater were obtained in some cases [Fig. 7 ▸(*a*)].

Na_4_Si_24_ and other clathrate formation have been studied in the Na_4_Si_4_ + Si system at HPHT conditions using electrical probing. Comparison of isopleth sections [Fig. 7 ▸(*b*)] at Na:Si = 1:5.5 (black color code) and Na:Si = 1:6 (red color code) illustrates typical information that in combination with *ex situ* XRD can be used to refine some equilibrium line, even if it is not possible to replace the *in situ* synchrotron data completely, especially for exact *p–T* positions of equilibria (Le Godec & Courac, 2021 ▸). This diagram shows the approximate domain of existence of three clathrates, Na_4_Si_24_, Na_8_Si_46_ (structure I) and Na_30_Si_136_ (structure II). At the same time, we should notice that Na_4_Si_24_ has the lowest Na content, and even a light excess of Na blocks the stability of this phase, and clathrate II forms instead. Kinetics also plays an important role, and at the present time the best conditions for crystal growth of Na_4_Si_24_ (up to 500 µm single crystals) were achieved by adjusting both thermodynamics (Na concentration, *p*, *T*) and kinetics (heating rate) (Guerette *et al.*, 2018 ▸). In principle, the calorimetric time–temperature–power data can be extracted from quantitative analysis of the time–power–resistance curve; however, only some limited achievements have been made so far from the methodological point of view (Geballe *et al.*, 2017 ▸).

## 3. Dense silicon allotropes

Common diamond structure of Si has an intermediate density (Table 3 ▸) between HP phases and open-framework structures (formally negative-pressure phases). Dense crystal forms can be directly obtained or crystallized from the melt at HP, but generally recovered phases are only metastable Si-III or HP compounds.

### 3.1. Direct pressure-induced transformations in silicon

#### 3.1.1. Si-I → Si-II: collapse and metallization

Si-I or diamond silicon has the lowest density among known packed arrangements of Si atoms in diamond structure, which has a high bulk modulus (low compressibility) and low thermal expansion as compared to other atomic arrangements (Table 2 ▸). Covalent Si—Si bonds are quite rigid and withstand pressure as high as ∼10 or even 15 GPa (never higher). Above this value, the volume collapses by ∼20% [Fig. 4 ▸(*a*)] with formation of Si-II with β-tin structure. Liquid Si is also denser than Si-I, which leads to the negative pressure slope of the melting curve up to ∼10 GPa (Bundy, 1964 ▸; Kubo *et al.*, 2008 ▸), similar to other materials with diamond and zinc-blend structure. It is important to note that the best samples of metastable Si materials are typically obtained at 12–15 GPa, *i.e.* where Si-II undergoes phase transformation into Si-XI or even to Si-V, so the underlying mechanisms and phase diagrams up to ∼15 GPa are of potential interest and will be considered here.

To get some insight into HP thermodynamics, some hydro­static data are usually needed. Analysis of Anzellini’s data on Si-I transformation in quasi-hydro­static He pressure medium (Anzellini *et al.*, 2019 ▸), sheds some light on the mechanism of the originally reported II→XI transformation (McMahon *et al.*, 1994 ▸). The transformation has pronounced second-order features, such as very close crystallographic density of both phases at the pressure of formation. At 300 K, the transformation seems to be slow, and a coexistence domain is often reported. Interestingly, the data indicate a continuous volume change with *ΔV* = 0 for all coexistence pressures, which is intrinsic to a second-order phase transition.

Atomic volume and unit-cell parameters (Fig. 4 ▸) are quite a natural way of presenting the HP phase transformations in Si that can be observed directly by *in situ* XRD or using alternative linear-sized change detection methods (not described here).

#### 3.1.2. Si-II → Si-XI → Si-V: symmetry lowering and order of transitions

It is thus generally believed that during compression at 300 K in quasi-hydro­static conditions, Si-I → Si-II transformation occurs at ∼11 (1) GPa. Si-II is a metallic form with tetragonal β-Sn structure and strong negative *ΔV* during this transformation allows it to be attributed to a first-order (or structurally discontinuous) transformation. Subsequent compression leads to Si-II → Si-XI transformation above ∼13 GPa. Si-XI has the orthorhombically distorted structure of Si-II and, according to the unit-cell parameters evolution can be a second-order phase transition (*i.e.* with *ΔV* = 0 or continuous transformation according to Landau classification). However, the data reported so far allow only the suggestion that *ΔV* < 0.6% [Fig. 4 ▸(*c*)], and higher-resolution data are required to resolve this issue, which is quite important for the topology of the Si phase diagram and consistency with theory. Unavoidable methodology error should be always considered, such as a small number of *hkl* reflections as compared to the number of unit-cell parameters. This may be crucial for some conclusions, and the data from some papers, taken as they are, may be contradictory, *e.g.* the *ΔV* at the critical point versus *ΔV* at equilibrium [Fig. 4 ▸(*c*)]. In fact, the critical point of unit-cell parameter *a* is at 13.5 GPa [Fig. 4 ▸(*b*)], while *V*_II_ = *V*_XI_ is at 13 GPa. One may expect that higher-pressure resolution is required to establish equilibrium points. HT may help since it is expected to render the transitions faster, and reduce stresses and the phase coexistence domain. The possibility of describing this transition in terms of phenomenological thermodynamics is a problem to resolve for the integration of these transformations into simulations of new materials. The question of coupled order parameters *a* and *b* (crystal unit-cell parameters), and *ΔV* (for CALPHAD methodology), as well as the relationship between critical parameters (*p*_cr_^*a*^, *p*_cr_^*b*^, *T*_cr_^*a*^, *T*_cr_^*b*^) is to be clarified in future experiments.

At highest pressure limit of our interest (above 15 GPa), relevant primarily for industrial applications, the Si-XI → Si-V transition occurs. Si-V has primitive hexagonal packing and is stable to very HPs, which is out of range of our interest for materials science purposes. Speculation on whether this transition is of first or second order exists, while experimental Δ*V* is much lower, if not zero, than most other phase transitions in silicon; and is of the order of magnitude of the statistical and systematic experimental errors. Table 3 ▸ presents the available-to-date parameters of the Kurakevych–Solozhenko equation of state fitted to *p–V–T* data (Kurakevych & Solozhenko, 2014 ▸), which is an analytical integrated form of the Anderson–Gruneisen equation (Anderson & Isaak, 1993 ▸).

#### 3.1.3. Recovery pathways: Si-III and Si-XII

**Figure 8:**
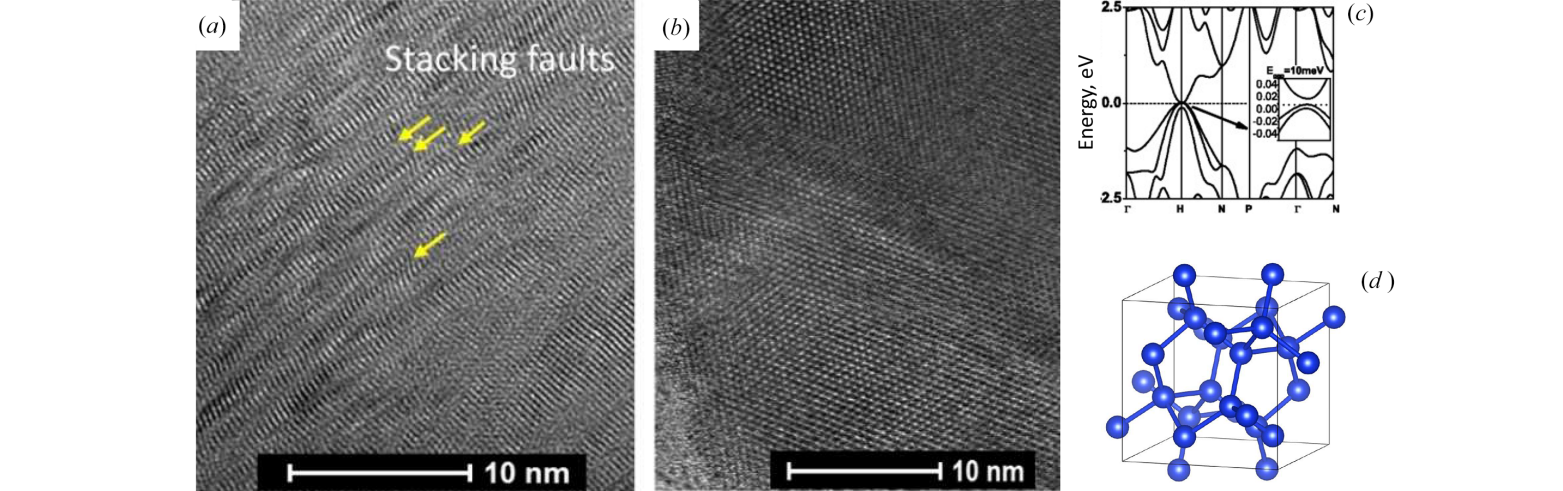
(*a*) High-resolution TEM image of Si-III grains obtained by direct phase transformations of pure Si (numerous stacking faults) and (*b*) by crystallization in the Na–Si system (zero stacking faults). (*c*) Electronic structure of BC8 silicon (Si-III). (*d*) Crystal structure of BC8 silicon (Si-III).

Fig. 5 ▸(*a*) shows the *in situ* mechanism of an archetypical HPHT Si synthesis experiment (Kurakevych *et al.*, 2016 ▸), observed as a sequence of direct phase transformation in Si; while on the right side, we provide raw data on the direct *in situ* observation of Na_4_Si_24_ formation in real time, as a sequence of chemical reactions, similar to those studied in intermetallic chemistry. Si-III can be recovered as a pure phase by quench, if the pressure drop (to ∼4 GPa) is sufficient enough. When quenched samples had pressure above 8 GPa, Si-II was generally observed in multianvil experiments, and it decomposed into Si-III and occasionally Si-XII, with high degrees of stacking faults and lower grain size [Fig. 8 ▸(*a*)]. The best Si-III samples with clearly distinguishable single-crystal domains above 10 nm were obtained by quench from the Na–Si system (Kurakevych *et al.*, 2016 ▸) [Fig. 8 ▸(*b*)]. The hydro­static conditions of such synthesis raise some doubts on the crucial role of shear stresses in BC8 phase formation, commonly suggested, and could support the existence of the Si-III stability domain in the phase diagram. At the same time, the pressure drop (a typical and irreproducible phenomenon that occurs on quench) should be considered as a part of the mechanism, and in our case the pressure drop to below 4 GPa (Kurakevych *et al.*, 2016 ▸) does not agree with pressures of ∼10 GPa for Si-III stability suggested by Blank & Estrin (2013 ▸). Si-XII recovery to ambient conditions in DAC experiments can be suggested from the EOS data plotted, but residual pressure is not precluded, and its possibility of being prepared in a large-volume sample should not be completely excluded. A recent example of ZnO shows that the geometry of the decompression may have a strong impact on nucleation and growth during reverse transformation, block the growth of low-pressure phase and, thus, favor the recovery of HP phases (Sokolov *et al.*, 2023 ▸).

### 3.2. Polytypism and hexagonal silicon

#### 3.2.1. Si 2H

Si-III upon heating passes into Si-IV. For a long time this allotrope was indexed as 2H according to identified main *hkl* reflections for wurtzite-type structure (Wentorf & Kasper, 1963 ▸). In addition, the experimental XRD intensities were completely different from the expected wurtzitic crystal structure, that was reported only once with experimental powder XRD intensities (Demishev *et al.*, 1996 ▸). The formal explanations were limited to the nanostructured nature of the sample and stacking faults; while neither of them allowed for a correct description. Generally, nanostructured wurtzitic or cubic phases show quite good agreement in terms of diffracted intensity, as observed for boron nitride of 3C and 2H polytypes, *i.e.* wBN (Kurakevych & Solozhenko, 2016 ▸) and cBN (Solozhenko *et al.*, 2012 ▸). The stacking faults are a typical qualitative explanation of this situation, however, they can be so significant (over 30%) that one could hardly talk about an 2H phase with 100% of formal hexagonality. Instead, the search among structural analogs of SiC polytypes or more usually called diamond polytypes, although carbon itself does not adopt most of them. It should be noted that Si-2H exists, and definitely differs crystallographically (by powder XRD) and spectroscopically (Raman) from HP Si-IV in LVP or by indentation (Sasidharan Nisha *et al.*, 2025 ▸).

#### 3.2.2. Si-4H

**Figure 9:**
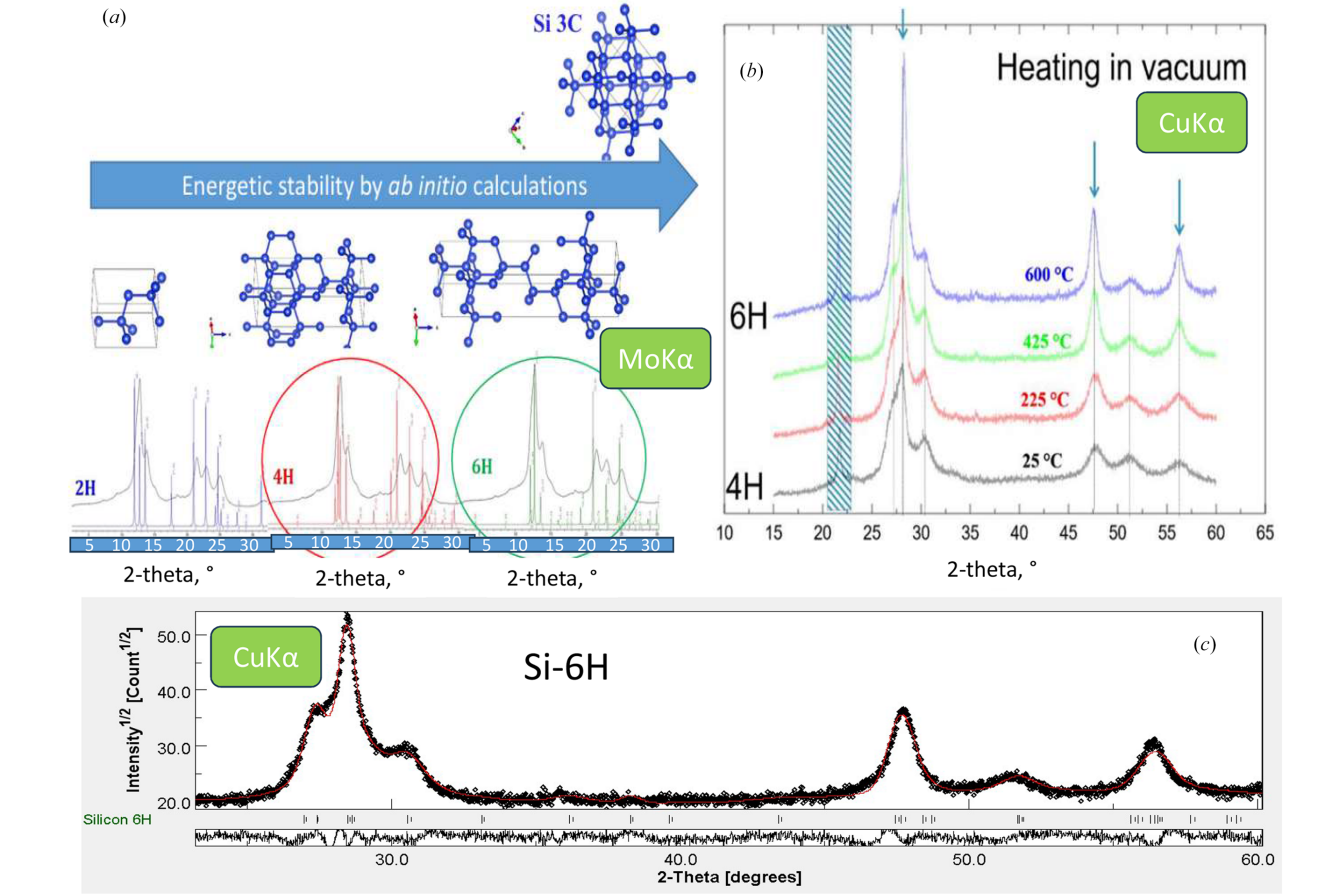
(*a*) Sequences of the evolution of Si-IV polytypes from least stable Si-2H (100% of hexagonality) to stable Si-3C (0% of hexagonality) and (*b*) *in situ* experimental observation of Si-4H to Si-6H transformation by X-ray diffraction during heating under vacuum. (*c*) Rietveld refinement of nanostructured Si-6H allotrope.

The attempt to resolve the crystal structure of Si-IV was successful in a combined powder XRD and TEM electron diffraction study (Pandolfi *et al.*, 2018 ▸). The correct intensities of *hkl* reflections were reproduced [Fig. 9 ▸(*a*)] and Rietveld refinement validated the 4H crystal structure model. Solid-state ^29^Si NMR confirmed this, although Raman spectra showed phonon density of states via structural disorder rather than individual modes of the 4H crystal structure. Later confirmation of Si-4H came from the observation of the Si_24_ to Si-4H transformation, which made it possible to observe well crystallized domains of Si-4H up to 5 µm (Shiell *et al.*, 2021 ▸).

#### 3.2.3. *In situ* synthesis of Si-6H

The sequence of increasing stability [Fig. 9 ▸(*a*)] for hexagonal polytypes of diamond silicon has been predicted by *ab initio* calculations (Raffy *et al.*, 2002 ▸). In our experiments, we observed Si-III → Si-IV(4H) transformation *ex situ* in the BN capsule (after synthesis of Si-III) (Pandolfi *et al.*, 2018 ▸). After the removal of residual stress (as a part of sample dispersion for TEM in liquid water), Si-IV(4H) → Si-IV(6H) has been observed *in situ* by XRD [Fig. 9 ▸(*b*)]. Si-6H shows similar strong photoluminescence as pure Si-IV(4H). Although, the results should be treated cautiously since the grain surface is most probably due to incomplete dehydration and partial oxidation of the surface of the starting Si-4H, even after 600°C treatment. The role of stress release under hydro­static conditions before subsequent treatment of HP phases is another topic to be explored. Fig. S1 shows a comparison of Rietveld refinement of both hexagonal phases. The Warren model allows an improvement of fitting quality.

#### 3.2.4. Role of stress, temperature and hydro­static environment

To further explore this domain, it is highly desirable to get more insight into phase transformations under various time-heating profiles [like for HP transformations in ZnO (Solozhenko *et al.*, 2011 ▸)], as well as time–pressure regimes of different techniques (Sokolov, 2023 ▸). For example, it has been noted that under hydro­static conditions, *e.g.* while quench (by switching off the power) from Na–Si melt or Si-II, Si-III forms without traces of Si-XII. The latter is believed to be common, but it was never observed in the best sintered high-purity samples. In fact, additional local Si-XII to Si-III transformation on complete decompression never improves mechanical and other functional properties, and thus the preferable mechanisms are those where its formation can be avoided.

### 3.3. Phase diagram of silicon

**Figure 10:**
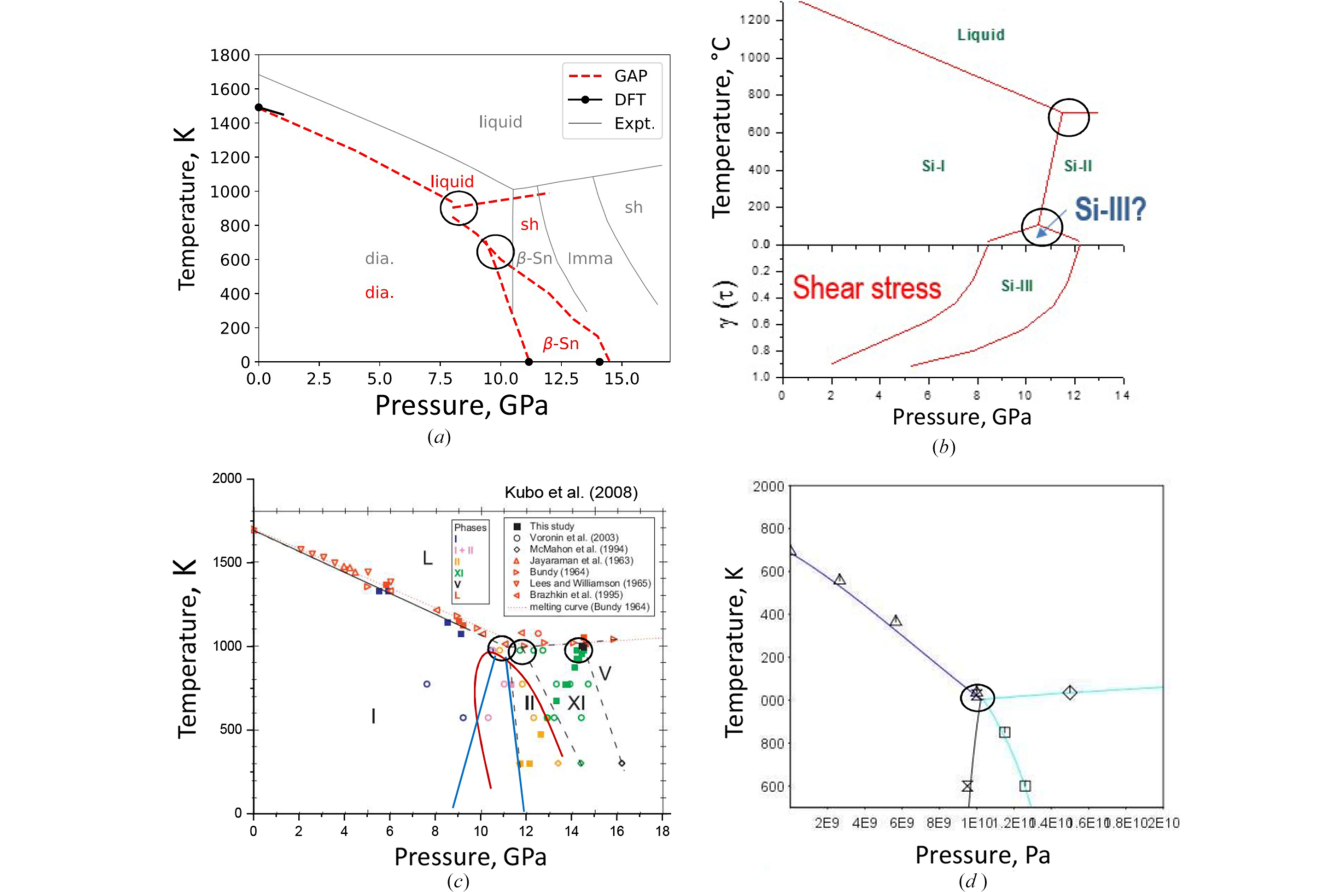
Phase diagrams of silicon by various methods. (*a*) *Ab initio* simulations (Bartók *et al.*, 2018 ▸), (*b*) Shear stress stabilization of Si-III (Blank & Estrin, 2013 ▸), (*c*) eye-guided lines to *in situ* points (Kubo *et al.*, 2008 ▸) (see text) and (*d*) eye-guided Thermocalc simulations of the domain of interest (one HP liquid with adjusted compressibility) (Courac, Le Godec *et al.*, 2025 ▸).

A phase diagram reflects the phase stability domain and is a primary guide for both experimental and computational design of advanced Si materials and gives the framework of more detailed mechanisms, *e.g.* some particularities are known for metastable and stable phase growth, typical for C, B and Si in pure states and in the presence of metallic solvents. Fig. 10 ▸ displays some phase diagrams that have been published in the literature. Fig. 10 ▸(*a*) shows a comparison of theoretical *ab initio* Monte Carlo simulations and machine learning, which often reproduce the general topology, but shows quite important discrepancy both in the terms of energy and sometimes even electronic structure of optimized crystal phases (*e.g.* neglecting free-electron contribution to heat capacity, *etc*.). The latter can be illustrated by the bandgap simulation: traditional DFT simulations give reasonable agreement for Si-I, but fail for Si-III. Its narrow bandgap observed experimentally can be reproduced only with Hartree–Fock exchange energy (Zhang *et al.*, 2017 ▸), and is often believed to be semi-metallic because of underestimated negative bandgap.

Systematic studies of Si behavior under shear stress and the formation of the Si-III phase [Fig. 10 ▸(*b*)] allowed for the extrapolation of the stability domain of the Si-III phase, which is believed to be intrinsically metastable by many researchers. The recovery of Si-III from quasi-hydro­static conditions at 4 GPa without a noticeable amount of shear strain is indicative that the topology pressure and/or the pressure of triple point Si-I/Si-II/Si-III, if it exists, should be different.

*In situ* data on melting of Si and HPHT phase transformations is shown at Fig. 10 ▸(*c*). The domains of coexistence of Si-I and Si-II and triple point Si-I/Si-II/L and Si-II/Si-XI/L are only guides for eye, and alternative tracing is possible using the same data, *e.g.* positive slope of Si-I/Si-II equilibrium or even triple point Si-I/Si-XI/L with Si-II domain stability enclosed in the solid state as a dome [Fig. 10 ▸(*c*)]. These issues are likely to be resolved only by means of thermodynamic simulations (Courac, Le Godec *et al.*, 2025 ▸) and HP calorimetry.

It is possible to formally include the available and sometimes contradictory equation of state data on HP allotropes of Si into the framework of CALPHAD methodology. Only one HP liquid was included. The thus-obtained phase diagram is presented in Fig. 10 ▸(*d*). It is mentioned here as a zero approximation for further refinements of potential importance for advanced Si materials design, so far under development for HP materials, such as diamond (Turkevich *et al.*, 2023 ▸) and boron (Courac, Turkevich & Le Godec, 2025 ▸). In perspective, kinetics data can be also incorporated into Thermocalc modules, not only for formal understanding of underlying mechanisms, but also for crystal growth and practical applications. Further refinement of this database is a challenge for exploratory HP materials science in the coming years.

## 4. Chemically assisted crystal growth in Na–Si systems

Solvent-based HPHT crystal growth of a precursor of Si_24_ has been realized and is a major conceptual achievement towards single-crystal growth of exotic silicon forms, comparable to artificial diamond synthesis using the solvent method. However, the pressure required is two times higher, ∼10 GPa, which imposes the questioning of simple analogies such as technological, thermodynamic and the nucleation and growth mechanisms at HPHT (Solozhenko *et al.*, 2002 ▸).

### 4.1. Open-framework and clathrate silicon structures

Silicon clathrates are intermetallic compounds of host–guest type, structural analogs of water clathrates (Cros & Pouchard, 2009 ▸; Kasper *et al.*, 1965 ▸). Some of them, such as Si_136_ or Si_24_, allow the extraction of intercalated atoms without destroying the covalent framework.

Open-framework clathrate allotropes of silicon are formally negative-pressure phases (Daisenberger *et al.*, 2010 ▸; Wilson & McMillan, 2003 ▸), and the most promising silicon structures predicted by *ab initio* evolutionary algorithms are of this kind (Wang *et al.*, 2014 ▸). The negative-pressure concept suggests that voids can represent the stability of states with positive *ΔV* formation, such as of a deintercalation reaction/void formation during mechanical negative tension.

Such crystal structures of Si can be obtained by removing the intercalated atoms (*e.g.* Na) encapsulated in cages (in the case of clathrate II Na_24_Si_136_ or Na_8_Si_46_) or channels (in the case of Na_4_Si_24_). The necessity of guest atoms to form the silicon framework has been discussed in previous works (Zwijnenburg *et al.*, 2010 ▸), their role consists of stabilizing the framework at HP from the thermodynamic point of view, due to negative *ΔV* of the intercalation reaction. Na atoms take a place in the crystal structure, giving rise to stable and well crystallized compounds.

In fact, framework structures with intercalated atoms often allow the system’s volume to increase, which renders them stable under HP (Kurakevych *et al.*, 2013 ▸). The covalent bonds of some frameworks can survive during removal of some types of intercalated atoms. This is generally the case with Na, as this alkali metal has small atomic size as compared to other metals that form such systems (K, Rb, Cs, Ca, Sr, Ba, I). Thus, the mechanism to understand is the formation (nucleation and growth) of such clathrates under HPHT conditions in the Na–Si system (similar to diamond crystallization in the presence of a solvent), and, at the same time, the mechanism of decomposition. Strictly speaking, HPHT samples of clathrates are better crystallized equilibrium phases, thus the variety of possible nucleation places is expected to be much lower as compared to chemical Na_4_Si_4_ decomposition synthesis (Song *et al.*, 2021 ▸).

Na_*x*_Si_136_ is a non-stoichiometric compound, ‘ideal’ stoichiometry at *x* = 24 (for a clathrate we adopt the coefficients in the chemical formula corresponding to unit-cell composition); *x* greater than 24 up to 30.5 corresponds to an HP phase denoted as HP-sII (Yamanaka *et al.*, 2014 ▸), while at ambient pressure in Ar or under vacuum sII with *x* from 0 to 24 forms (Cros & Pouchard, 2009 ▸; Kasper *et al.*, 1965 ▸). The fact that the sII phase with *x* = 30.5 is thermodynamically stable only at HP, while *x* = 0 is stable only at negative pressure together with the wide stoichiometry range of phase existence, suggests the existence of *x*^eq^ corresponding to the thermodynamic stability at ambient pressure. The *p–T–x* range of thermodynamic stability for sII/HP-SII has not been studied so far, even if it seems possible since unit-cell parameters are a fingerprint of such phases, and from a functional point of view they can be promising by Mott transition between semiconductor and metal state at some relatively large *x* (*x* > 3). The time scale of heating is another important factor that can vary from a couple of hours to a couple weeks in a quite narrow temperature (713–733 K) (Song *et al.*, 2021 ▸) range. *In situ* removal of Na from HP sII Na_30.5_Si_136_ passes primarily by removal of the second Na from the largest cages, and after the full removal of Na just like for conventional chemical synthesis. *In situ* XRD patterns collected during Si_136_ synthesis under vacuum from this HP clathrate were previously reported by Le Godec & Courac (2021 ▸).

Na_*x*_Si_46_, the clathrate of the sI structure, adopt mainly *x* close to 8 (the lowest reported value 6) and have never been observed as pure Si with *x* ∼ 0. It has been established that sI is an HP phase stable above 3 GPa and up to 8 GPa (Kurakevych *et al.*, 2013 ▸). So far no removal of the intercalated atom from structure I clathrate and formation of Si_46_ has been reported. Clathrate sodium silicide with type I structure, obtained without HP techniques, can easily decompose into clathrate type II during heating, which has previously been noted (Cros *et al.*, 1970 ▸). Decomposition of well crystallized HP species have not been studied so far.

In the case of the channel clathrate structure of Na_*x*_Si_24_, only phases with stoichiometries *x* = 4 or *x* ∼ 0 are known (Guerette *et al.*, 2018 ▸; Kim *et al.*, 2015 ▸; Kurakevych *et al.*, 2013 ▸). Long storing of Na_4_Si_24_ leads to sodium escape from the lattice, and its hydrolysis on the surface with air humidity. Anyway, heating under vacuum remains the method of choice to produce the best samples of Si_24_ (Kim *et al.*, 2015 ▸).

### 4.2. Phase diagrams and thermodynamics of the Na–Si system

#### 4.2.1. Ambient-pressure Na–Si diagram

**Figure 11:**
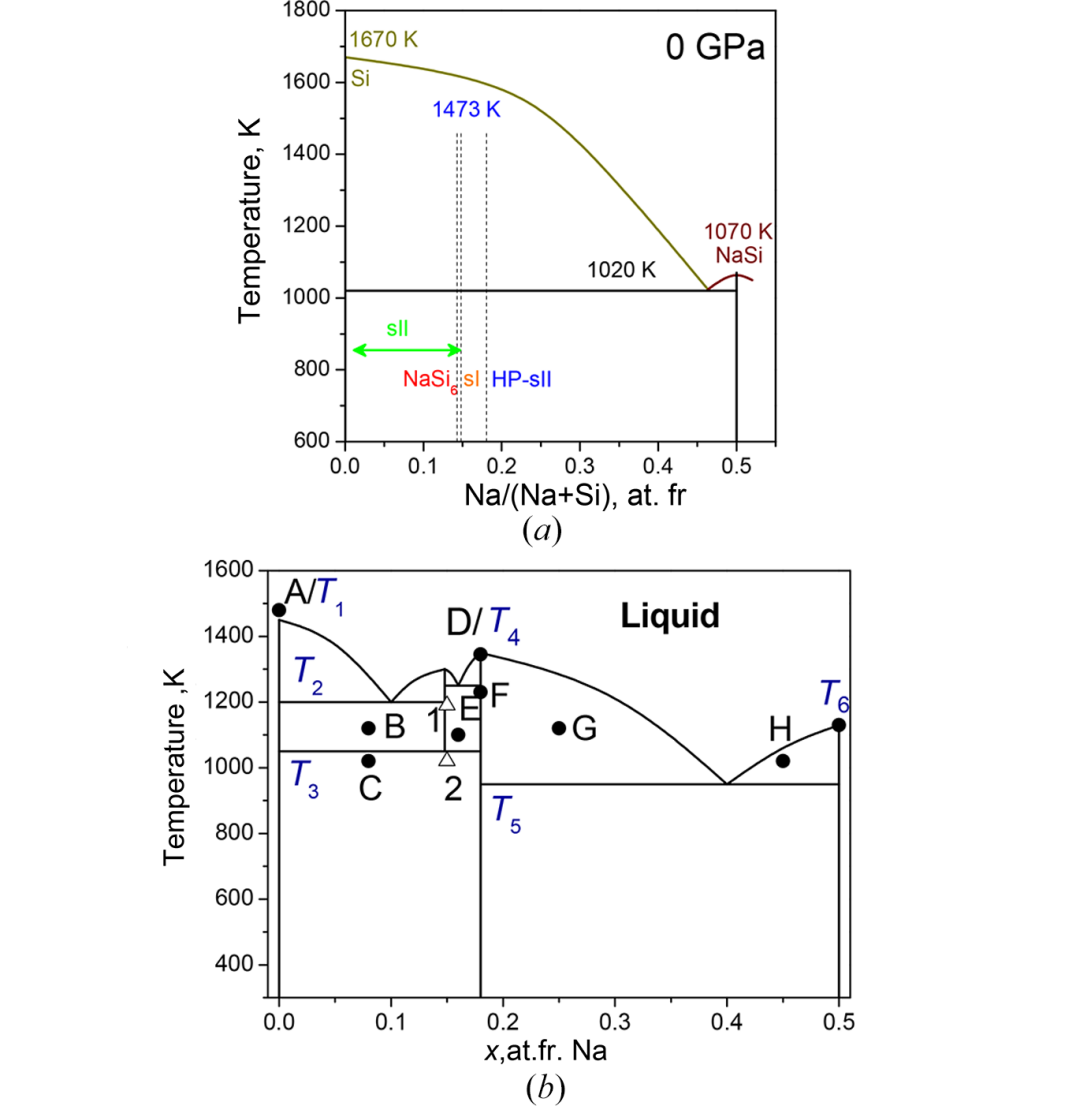
(*a*) Binary phase diagram of the NaSi–Si system showing the presence of Na_4_Si_4_, which forms eutectic equilibria with Si (Morito *et al.*, 2009 ▸). (*b*) Phase diagram of the NaSi–Si system at 4 GPa with two clathrates, Na_8_Si_46_ (sI), and Na_30.5_Si_136_ (sII-HP). The points A to H are explained in Table 4 ▸.

The binary phase diagram of the NaSi–Si system at ambient pressure is presented in Fig. 11 ▸(*a*) (Morito *et al.*, 2009 ▸). In this system, the possibility of growing Si-I single crystals from liquid Na–Si solutions was also demonstrated. At HPHT, Si-II crystal growth should be possible (Kurakevych *et al.*, 2016 ▸). This phase diagram at 0.1 MPa was constructed using differential thermal analysis and XRD on samples prepared with various compositions.

#### 4.2.2. HP phase relations

The unique ambient-pressure compound that participates in the equilibria in whole Na–Si is Na_4_Si_4_, which forms a eutectic equilibrium with Si-I (and Na, not discussed here). The Na_4_Si_4_ compound exhibits congruent melting at 1071 K and this melting temperature increases with pressure (Courac *et al.*, 2019 ▸). Since the melting temperatures of Si and Na_4_Si_4_ have opposite pressure slope, at 4 GPa the phase diagram is supposed to include two compounds, sI and HP-sII [Fig. 11 ▸(*b*)]. At the same time, one important issue should be emphasized. In fact, HP-sII, or Na_*x*_Si_136_ with *x* = 30.5, is a high-pressure phase, present on the phase diagram at HP, while at *x* = 0, it is definitely a negative-pressure phase. The continuous change of Gibbs energy of the atom-vacancy (Na-Na2-V) solid solution with the sII crystal structure is the most probable model that allows us to suggest the existence of equilibrium composition *x*^eq^ at ambient pressure, at least at low and room temperature.

#### 4.2.3. Stability domains of clathrates sI, sII and Na_4_Si_24_

In previous papers, we described *in situ* and *ex situ* experimental results. To construct an experimental phase diagram, it is important to emphasize that the compounds participating in the equilibria, *i.e.* Na_4_Si_4_, Na_8_Si_46_ (sI) and Na_30.5_Si_136_(sII-HP), are stoichiometric and do not form solid solutions. sII-HP coexists with Si-I at low temperatures, while sI becomes more stable at higher temperatures in the case of excess Si. Excess Na makes sII-HP stable up to the melting temperature. Previous reports suggest that *ΔV* of melting be close to 0 (Courac *et al.*, 2019 ▸), thus with a weak d*T*/d*p* slope of the melting curve. Rapid quenching of a stoichiometric liquid results in the crystallization of sII-HP, indicating that the melting of this phase is congruent. Some experiments show the coexistence of sI and sII-HP with the absence of Si-I. Fig. 11 ▸(*b*) represents the tentative phase diagram compatible with all the mentioned experimental observations (Tables 4 ▸ and 5 ▸).

### 4.3. Nucleation and crystal growth kinetics

As for the nucleation and growth of Si at HPHT conditions, the key contribution in the field of exotic Si phase single-crystal growth concerns Na_4_Si_24_ and the related Si_24_ allotrope. Such growth is a technological analog of the solvent-assisted growth of diamond. As for direct transformation single-crystal growth, no success is known so far. At the same time, the direct transformation of Si_24_ to Si-IV (4H polytype) is possible and produces large single-crystal domains, up to 5 µm (Shiell *et al.*, 2021 ▸). This result can encourage future works tailoring various forms of hexagonal silicon.

So far, more detailed studies are required of the kinetic factors influencing silicon transformations, for example, to fully understand how experimental conditions (heating rates, presence/absence of pressure medium, stress, impurities) affect kinetics and transformation outcomes. No linear growth kinetic constants or activation energies of direct phase transitions are available so far.

#### 4.3.1. Solvent-assisted growth of Na_4_Si_24_

The time factor, in terms of the heating rate and heating profiles, has been taken into account in a previous study (Guerette *et al.*, 2018 ▸). The kinetics of Na_4_Si_24_ nucleation and growth are sensitive to synthesis conditions, particularly pressure, temperature, heating rate, and compositional ratios (Guerette *et al.*, 2018 ▸). Optimal growth occurs around 9.0 GPa in the Na+Si system (flat interface), with pressures below 8.0 GPa generally insufficient and higher pressures offering little improvement. Temperature strongly influences phase yields, peaking near 1123 K, beyond which Na_4_Si_24_ melts incongruently, ceasing formation. Rapid heating (∼1.7 K s^−1^) effectively suppresses competing phases like the sII clathrate, promoting direct nucleation of Na_4_Si_24_, whereas slow heating rates (0.2 K s^−1^) initially favor sII formation, with subsequent conversion to Na_4_Si_24_ upon Si-I depletion. Na_4_Si_24_ exhibits notable phase competition with sII clathrate and Si-I, requiring precise control of the Na:Si ratio (∼1:6) to maximize its yield. Single crystals exceeding 500 µm have been synthesized using single-crystalline diamond cubic Si wafers and elemental sodium, leveraging epitaxial coherency, particularly between the Na_4_Si_24_ {113} and Si-I {111} planes, facilitating enhanced nucleation and crystal growth. The structure features exceptionally high sodium mobility, verified via electron microscopy and electrical measurements, facilitating sodium removal and conversion to Si_24_. Short thermal cycles near melting conditions (∼1050–1150 K) further encourage significant grain growth without decomposition, highlighting kinetic sensitivity and offering practical strategies for scalable synthesis.

#### 4.3.2. Expected role of heating rate, stoichiometry and epitaxy

Investigating the kinetic of direct phase transformations under HPHT conditions remains challenging, although early *ex situ* studies have already been carried out by Bundy (1964 ▸). Synchrotron radiation allows more reliable kinetics data, taking into account diffusion, on solvent-assistant diamond (Solozhenko *et al.*, 2002 ▸) and direct phase transformations (Kurakevych & Solozhenko, 2016 ▸; Solozhenko *et al.*, 2011 ▸). Kinetic curves can be fitted to the adapted Avrami equation (Kurakevych, 2007 ▸) and provide a reliable picture of nucleation and growth when time–temperature profiles are taken into account (Solozhenko *et al.*, 2011 ▸). In some cases, crystallographic features of nucleation can be revealed directly via *in situ* XRD (Solozhenko & Kurakevych, 2005 ▸), however it is not, generally, the case with Si. In the case with Si, both direct transformation and solvent-assisted growth are relevant to exotic Si form synthesis. However, no kinetic curves have been analyzed so far.

The epitaxial formation of thin films of clathrate-I Si_4__6_, a hypothetical allotrope, can be achieved by removing intercalated metal from Ba_8_Si_46_ through electron-beam heating of individual grains (Zhou *et al.*, 2025 ▸). The possibility of scaling up this process with conventional thermal heating is still questionable.

## 5. Modeling and integration

Here we will explicitly compare consistency and discrepancies between theoretical and experimental data (see Table 3 ▸). When analyzing bibliography, it is crucial to make a clear distinction between experimental findings and theoretical predictions or expectations. Most of the facts described in this review are derived from experimental observation. As it was mentioned above, the data accuracy and reproducibility, particularly in terms of pressure, is comparable (fortunately, smaller) with the domain of existence itself, particularly where Si-II, Si-XI and precursor Na_4_Si_24_ are concerned.

### 5.1. Computational approaches: *ab initio* and CALPHAD

A number of *ab initio* studies are available, for example, Alfè *et al.* (2004 ▸) and Bartók *et al.* (2018 ▸). *Ab initio* phase diagrams reproduce general features observed experimentally [Fig. 8 ▸(*a*)]. However, the exact triple point in *p–T* parameters can hardly be predicted with reasonable accuracy, and therefore, the derived mechanism can be unrealistic. For example, open-framework Si, desired for its direct bandgap, as well as some negative-pressure phases and corresponding phase diagrams have been reported (Wilson & McMillan, 2003 ▸). However, the real mechanism is related to the Na–Si binary phase diagram, competing nucleation and growth of Si clathrates at HP, and kinetics of subsequent thermal decomposition.

Another example is HP phases of Si, which all have features of continuous second-order phase transitions. For example, Si-XI exhibits higher compressibility compared with Si-II (in the 13.5 to 16.5 range at least), aligning well with the formation of a phase through second-order phonon mode softening. Predictions for this behavior were made by the calculation of phonons during the transition and it suggests that the II→XI transition is second order, while the XI→V transition is most probably first order (Gaál-Nagy & Strauch, 2006 ▸). Further supporting evidence is provided by Needs and Lewis (Lewis & Cohen, 1993 ▸; Needs & Martin, 1984 ▸), who proposed a continuous deformation from II→XI→V based on enthalpy considerations, with Needs noting that Si-II has soft phonons at the transition. Other authors suggest that the II→XI transition could be second order in line with Landau theory. Lastly, the bulk modulus of Si-II is predicted to be 20–50% higher compared with Si-I, which aligns with *in situ* experiments. The possibility of kinetic factors of hysteresis and coexistence domain should be also examined in future experiments.

ThermoCalc phenomenological simulations using CALPHAD methodology were performed for Si at HPHT conditions, while the phase diagram thus obtained reproduces the *ab initio* phase diagram that explores geological pressures rather than experimental one (Brosh *et al.*, 2007 ▸). It is a good starting point for HP Si advanced materials design. It remains an open crystallographic problem of phenomenological consideration of second-order phase transition in non-magnetic material, which is of both fundamental and applied interest. Unit-cell parameters *a* and *b* seem to be appropriate natural 2D order parameters in the field of strain. In quasi-hydro­static conditions one can hope to reformulate it in terms of volume (1D order parameter) and pressure. This reformulation seems crucial for including HP Si phases into such simulations.

As for clathrates, their relative stability has been estimated by Kurakevych *et al.* (2013 ▸). Na_4_Si_24_ is predicted to be stable above 6 GPa. Only a narrow *p–T* domain of its stability has been experimentally observed between 8 and 10 GPa at ∼1000 K. The competition of crystallization mechanisms of three clathrates, sI, HP-sII and Na_4_Si_24_, is the key factor that often renders the synthesis result unpredictable. HP-sII has a very large domain of thermodynamic stability and suppression of its nucleation and growth is crucial point for successful synthesis of Na_4_Si_24_. *Ab initio* simulation predicts the thermodynamic stability of clathrate II at low temperature, which is in good agreement with experiment (Kurakevych *et al.*, 2013 ▸).

### 5.2. Unified transformation pathways

**Figure 12:**
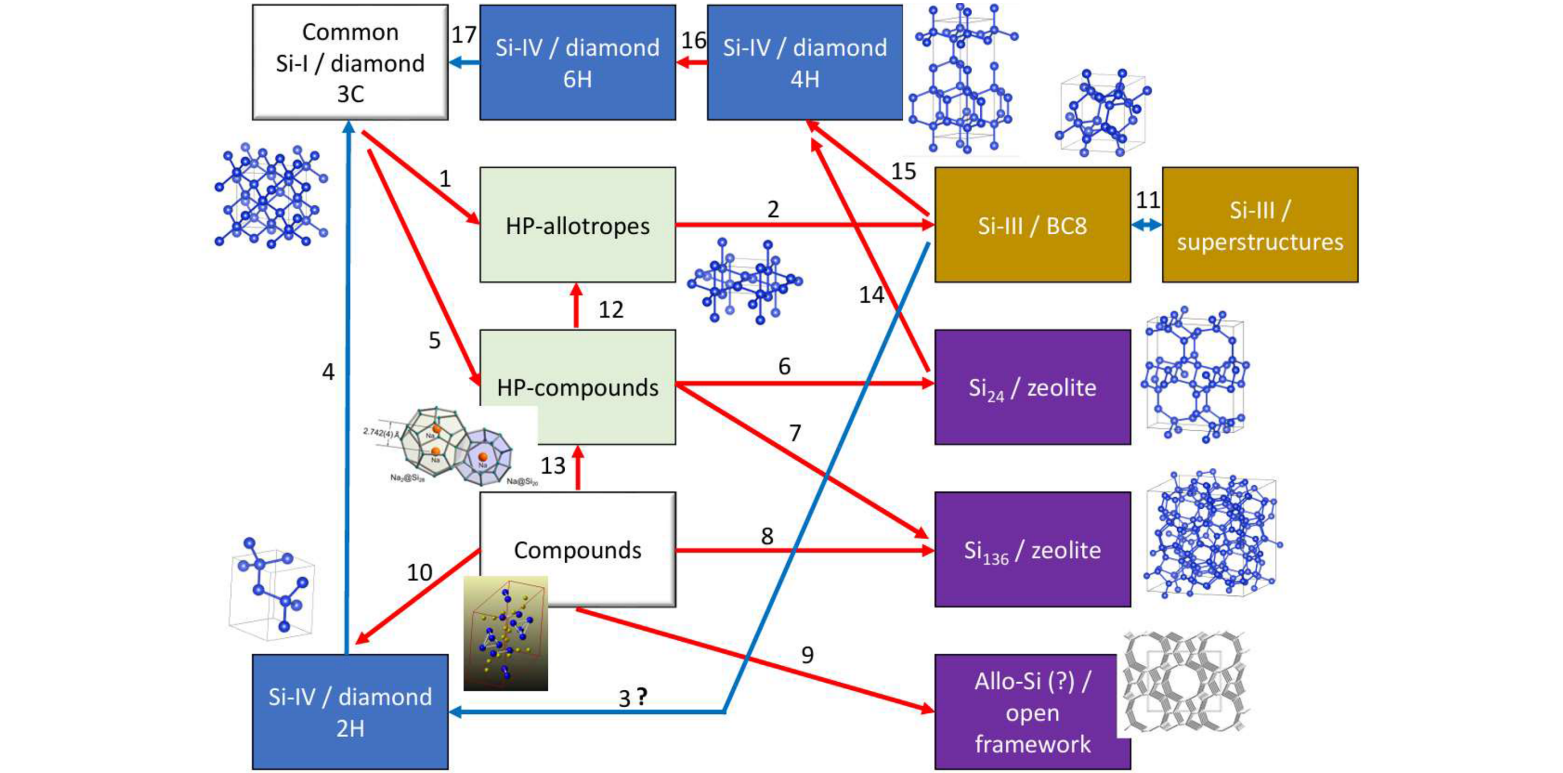
Transformation pathways between Si allotropes. The arrows indicate the possible transformation pathways between allotropes, either direct or chemically assisted.

Most silicon allotropes are mutually connected, via direct transformations or chemical routes (Fig. 12 ▸). This fact implies multiple possible mechanisms and diversity of possible materials. Table 6 ▸ shows the references for each arrow in Fig. 12 ▸, most of these transformations are not connected directly to Si-I. These transformations definitively play an important role in comprehending the mechanism of nanostructuration, as well as that of the growth of large crystals, and most of them can be reliably studied using advanced time and space resolution *in situ* XRD techniques.

## 6. Outlook and perspectives

Phase transformation mechanisms control crystal growth at HP. HP can be applied to conventional diamond Si with a wide range of experimental techniques, and initiates the rich diversity of silicon allotropes that form via synthesis in condensed media, notably within the Si and Na–Si systems. HP synthesis techniques, supported by advanced *in situ* methodologies, have revealed novel transformation mechanisms and phase relationships previously inaccessible through conventional methods. Most phases are intrinsically metastable, still, some of them show quite high thermal stability (to ∼600°C) that, combined with their structural versatility, is promising for new or ‘known-but-scaled’ silicon-based materials exhibiting desired functional properties, particularly in advanced photovoltaic applications.

The transition pathways, such as those from Si-I to dense allotropes like Si-II, Si-III, and ultimately Si-IV polytypes, highlight the critical role of thermodynamics and kinetics in governing phase stability and transformations. Particularly notable is the observed direct or quasi-direct bandgap characteristics in allotropes such as Si-IV and silicon clathrates (Na_8_Si_46_, Na_30_Si_136_, and Na_4_Si_24_), positioning these phases as promising candidates for next-generation solar technologies aiming to surpass the Shockley–Queisser limit.

Emerging results from *ab initio* calculations, structural optimizations, and advanced predictive models complement experimental findings by providing deeper insight into the energetics, structural configurations, and stability domains of silicon phases. Such computational tools have become indispensable for predicting and validating novel phases prior to experimental synthesis, thereby, streamlining the discovery process.

Future research directions will inevitably focus on refining synthesis routes to achieve larger crystal sizes, enhanced phase purity of nanostructured bulks, and improved reproducibility. Despite the fact that many potential industrial applications are possible (Na–Si, Ba–Si, *etc*.), no reliable database for metal–Si systems for CALPHAD methodology is available. Further investigations into open-framework structures could expand the range of available negative-pressure silicon phases, enabling broader exploration of low-density materials with tunable electronic properties. Moreover, extending *in situ* large-volume studies with complementary characterization techniques, such as electron microscopy, spectroscopy, and electrical measurements, will be essential for a comprehensive understanding of the complex interplay between pressure, temperature, and chemical environment in shaping silicon allotropes.

Finally, the synergy between experimental advancements and theoretical predictions holds significant promise for discovering and optimizing silicon allotropes with tailored functionalities. Continued collaborative efforts across experimental and computational disciplines will undoubtedly lead to novel silicon-based materials that meet critical technological demands, especially in energy conversion, storage, and semiconductor technologies.

## Supplementary Material

Crystal structure: contains datablock(s) Silicon-6H. DOI: 10.1107/S2052520626004026/tq5036sup1.cif

Section S1, Table 1, Figures S1-S3. DOI: 10.1107/S2052520626004026/tq5036sup2.pdf

CCDC reference: 2547842

## Figures and Tables

**Table 1:** *In situ* techniques: resolution, quantities measured, and derived properties

*In situ* method and setup	Typical resolution	Measured quantities	Derived material properties	Pressure and temperature range
Synchrotron XRD (Paris–Edinburgh press)	Spatial: ∼10–50 µm (up to 1–2 mm for reaction volume); temporal: ∼10 s	Diffraction pattern†	Atomic volume, phase identification, crystal structure, unit-cell parameters	0–25 GPa; 300–2500 K
Synchrotron XRD (diamond anvil cell: DAC)	Spatial: ∼10–30 µm; temporal: ∼1–5 s	Diffraction pattern†	Unit-cell parameters, phase transition kinetics, anisotropic strain	0–150 GPa; 300–3500 K
Synchrotron HP-HT XRD (multianvil)	Spatial: ∼10–50 µm (up to 1–2 mm for reaction volume); temporal: ∼10–60 s	Diffraction pattern†	High-volume averaged structure, phase boundary mapping	0–30 GPa; 300–2500 K
Synchrotron HP-HT-stress XRD (RoToPEc and RDAC)	Spatial: ∼10–30 µm; temporal: ∼1–5 s	Diffraction pattern	Unit-cell parameters, phase transition kinetics, anisotropic strain	0–20 GPa; 300–1600 K
Electrical resistance (DAC)	Temporal: ∼1 ms–1 s	Resistance, I–V characteristics	Melting detection, metallization, bandgap estimation	0–25 GPa; 300–1000 K
Electrical resistance (large-volume press)	Temporal: ∼1 ms–10 s	Resistance versus *T*, real-time monitoring	Electronic transitions, clathrate stability, kinetics	0–20 GPa; 300–2500 K
*In situ* calorimetry (DAC)	Temporal: ∼0.1–1 s	Power–time signal, Δ*T*	Onset of transformation, enthalpy estimation	0–20 GPa; 300–1000 K
*In situ* calorimetry (large-volume press)	Temporal: ∼1–10 s	Voltage–temperature jump	Phase transition heat, temperature calibration	0–20 GPa; 300–2200 K
Hardness by conductive diamond indenter (HP-DAC)	Spatial: ∼5 µm; temporal: static	Penetration depth	Hardness, yield strength, plasticity onset	0–25 GPa; 300–1000 K
Optical spectroscopy (DAC/transparent anvils)	Temporal: real-time	Reflectance/transmittance spectra	Bandgap variation, metallization, thermal emission	0–100 GPa; 80–1500 K
Additional methods not covered in this study (Raman, XANES, neutron diffraction)	Variable	Vibrational modes, absorption edges, scattering	Bond order, oxidation state, site occupancy	0–100 GPa; 100–2000 K

† Various, but not exhaustive possibilities are 1D intensity versus 2θ (monochromator beam); intensity versus photon energy (fixed angle, energy-dispersive beam); 2D image plate data (monochromator beam); 2D image plate data with energy dispersive beam (non-trivial and rear).

**Table 2:** Summary of silicon allotropes up to 15 GPa Data principally from references: Minomura & Drickamer (1962 ▸), McMahon *et al.* (1994 ▸), Wentorf & Kasper (1963 ▸), Hauge *et al.* (2015 ▸), Pandolfi *et al.* (2018 ▸), Kasper *et al.* (1965 ▸), Kim *et al.* (2015 ▸), Shiell *et al.* (2021 ▸).

Allotrope	Structure/type	Stability at ambient conditions	Bandgap (eV)	Synthesis route	Key features/applications
Si-I (Si-3C)	Diamond cubic (A4)/3C diamond polytype	Stable	1.12 (indirect)	Conventional CVD/crystal growth	Baseline for all Si materials; photovoltaic benchmark
Si-II	β-tin (A5)	Unstable at 300 K, metastable at 100 K; transforms on decompression	Metallic	HPHT (>11 GPa)	Intermediate phase; precursor to Si-III
Si-III (BC8)†	Cubic body-centered (BC8)	Metastable; quenchable	∼0.03 (narrow-gap)	Direct HP transformation or Na-assisted synthesis	Dense structure; high hardness
Si-IV (Si-4H)	Hexagonal polytypes among 2H / 4H / 6H	Metastable	1.2	Heat-treated Si-III or Na-assisted methods	Direct bandgap; promising for photovoltaics; photoluminescence (PL)
Si-XI	Orthorhombic	Metastable at ambient pressure	Metallic	HPHT (∼13–16 GPa)	Intermediate phase (Si-II → Si-XI → Si-V); second-order transition
Si-V	Primitive hexagonal (A_f_)	Stable only at very HP	Metallic	Compression >15 GPa	Precursor to Si-III
Si-XII (Si-R8)	Rhombohedral distortion of BC8	Metastable	∼0.3–0.5 (estimate)	Shock/strain-induced or decompression	Accompanies Si-III in rapid decompression
Si_136_ (clathrate-II Si)	Face-centered cubic, large cages	Metastable (*x* = 0); stable as Na_*x*_Si_136_	∼1.9–2.1 (direct)	HPHT synthesis with Na, followed by Na removal	Low density; strong PL; negative-pressure phase
Si_24_	Orthorhombic clathrate, 1D channels	Metastable (can be recovered)	∼1.3–1.5 (direct)	Na_4_Si_24_ → Si_24_ via vacuum heating	High sodium mobility; channel framework; PL active
Si_46_ (clathrate-I Si)	Cubic (sI-type)	Hypothetical as pure Si	Predicted ∼1.9 (direct)	Ba_8_Si_46_ → Si_46_;‡ metastable epitaxial film on nanograins	Framework known but Si_46_ not yet isolated
Si amorphous (a-Si)	Disordered network	Metastable	∼1.6–1.8 (indirect)	Laser ablation, deposition	Used in thin-film PV, precursor for HP transformations
Predicted phases	Various; mostly of low-density, high-symmetry	Not synthesized	From 0 up to 2.5 (direct)	*Ab initio* predictions, machine learning	Potential targets for direct-gap Si design

† Recoverable phase(s) as mm-sized species from HP or HPHT experiments phases

‡ Zhou *et al.* (2025 ▸).

**Table 3:** Structural, thermal, mechanical, and density data of silicon allotropes up to 15 GPa Data principally from references: Anzellini *et al.* (2019 ▸); Kurakevych *et al.* (2015 ▸); Beekman *et al.* (2022 ▸). Unit-cell parameters and density reported at lowest pressure available, at 0.1 MPa and 300 K if not indicated otherwise.

Allotrope	Space group	Unit-cell parameters (Å)	Density ρ (g cm^−3^)/*p–T* conditions	Bulk modulus, *B*_0_ (GPa)	*B*_0_′ (∂*B*/∂*P*)	Thermal expansion coefficient (×10^−6^ K^−1^)	Hardness (GPa)
Si-I (3C)	*Fd* 3 *m*	*a* = 5.431	2.330	101.5	3.43	2.6	12.0
Si-II†‡	*I*4_1_/*amd*	*a* = 4.760, *c* = 2.636	3.123 (0.1 MPa, 100 K)	80–150	4§	Unknown	∼7–10¶
Si-III (BC8)	*Ia* 3	*a* = 6.6276	2.560	120 (10)††	4	Unknown	17.1
Si-IV (4H)	*P*6_3_*mc* (2H/4H/6H)	*a* = 3.837, *c* = 12.520 (4H)	2.350	100§	4§	6 (Table S1)	11.2 (2)
Si-XI†	*Cmcm*	*a* = 4.737, *b* = 4.479, *c* = 2.552	3.440‡‡	45	4	Unknown	∼7–10¶
Si-V	*P*6/*mmm*	*a* = 2.553, *c* = 2.382	3.470 (15.4 GPa‡‡)	95	4.6	Unknown	∼7–10¶
Si-XII (R8)	*R* 3	*a* = 5.720, α = 109.95	2.592 (2 GPa††)	96 (5)††	4	Unknown	17.1
Si_136_	*Fd* 3 *m*	*a* = 14.62	2.030	90	5.2	2.5	∼7–10†
Si_24_	*Cmcm*	*a* = 3.8246, *b* = 10.700, *c* = 12.648	2.163	90§	4§	2.6§	∼7–10†
Si_46_	*Pm* 3 *n*	*a* = 10.11§	2.088§	90§	4§	Unknown	∼7–10†
a-Si	Disordered		2.29	Unknown	Unknown	Unknown	∼9–12
Predicted phases	Variable	Variable	2.0–2.4	Unknown	Unknown	2.6§	∼7–10†

† Extrapolation of HP data.

‡ Jamieson (1963 ▸).

§ Semiempirical estimation.

¶ Estimation using thermodynamic model of hardness of metals (Mukhanov *et al.*, 2008 ▸).

†† Piltz *et al.* (1995 ▸).

‡‡ McMahon *et al.* (1994 ▸).

§§ Estimation using thermodynamic model of hardness of semiconductors (Mukhanov *et al.*, 2009 ▸).

**Table 4:** Experimental support for the Na–Si phase diagram at 3–6 GPa Explanations to experimental points in Fig. 11 ▸(*b*).

Notation	Phase stability domain†	*P* (GPa)	*T* (K)	Remarks	References
A: T1	Liquid Si	4	1480	Experimental, *in situ* XRD	Kubo *et al.* (2008 ▸)
B†	Si + sI	3	1120	Experimental, *ex situ*	Our experiments, unpublished
C†	Si + sII	5	1020	Experimental, *ex situ*	
D: T4	Liquid sII	6	1345	Experimental, *in situ* XRD	Kurakevych (2016 ▸)
E†	sI + sII	5	1100	Experimental, *ex situ*	Jouini *et al.* (2016 ▸), Kurakevych *et al.* (2013 ▸), Yamanaka *et al.* (2014 ▸)
F†	sII	5	1230	Experimental, *ex situ*	Jouini *et al.* (2016 ▸)
G	sII + Na_4_Si_4_	4	1120	Experimental, *in situ* XRD	Jouini *et al.* (2016 ▸)
H†	Na_4_Si_4_ + sII	3	1020	Experimental, *ex situ*	Kurakevych *et al.* (2013 ▸), Yamanaka *et al.* (2014 ▸)
T6	Liquid Na_4_Si_4_	4	1130	Experimental, *in situ* electrical	Courac *et al.* (2019 ▸)
T6	Liquid Na_4_Si_4_	4	1130	Experimental, *in situ* electrical	Courac *et al.* (2019 ▸)

† These domains were principally explored by recovery experiments. The phase composition was established by *ex situ* XRD.

**Table 5:** Phase-equilibrium temperatures observed in the Na–Si system at 4 GPa Explanations to equilibrium temperatures in Fig. 11 ▸(*b*).

Notation	Phase equilibrium	*T* (K)	Remarks	References
T_1_	Si-I ↔ Si-L	1480		Kubo *et al.* (2008 ▸)
T_2_	Si-I + sI ↔ L	1200	Hypothesis; ‘contact’ eutectic melting	
T_3_	Si-I + sII ↔ sI	1050	Pseudo-polymorphism of clathrate(s)	Kurakevych *et al.* (2013 ▸), Yamanaka *et al.* (2014 ▸)
T_4_	sII ↔ L	1345	*In situ* XRD at 6 GPa	Kurakevych (2016 ▸)
T_5_	Na_4_Si_4_ + sII ↔ L	950		
T_6_	Na_4_Si_4_ (s) ↔ L	1130	L = Na_4_Si_4_(liquid); *in situ* electrical	Courac *et al.* (2019 ▸)

**Table 6:** Transformation pathways between Si allotropes Explanations to Fig. 12 ▸.

Arrow(s)	Comments	Reference(s)
1 / 2	Formation of Si-III is also possible without intermediate HP phases under shear stress	Wentorf & Kasper (1963 ▸), Yesudhas *et al.* (2024 ▸)
3	Originally suggested, but not confirmed in crystallographic XRD and TEM studies	Pandolfi *et al.* (2018 ▸)
4 / 17	Proved for both LVP and indentation phases.	Brazhkin *et al.* (1992 ▸), Ge *et al.* (2004 ▸), Sasidharan Nisha *et al.* (2025 ▸)
5	Some HP compounds can serve a precursor for Si allotropes, line Na_4_Si_24_ (or NaSi_6_)	Kurakevych *et al.* (2013 ▸)
6	Na can be easily removed, without disturbing Si framework of compound Na_4_Si_24_	Kim *et al.* (2015 ▸)
7	Na can be easily removed, without disturbing Si framework of compound Na_30.5_Si_24_	Yamanaka *et al.* (2014 ▸)
8	Na removal induces the polymerization of Si_4_^4−^ anions of compound Na_4_Si_4_ giving rise to clathrate Si frameworks	Kasper *et al.* (1965 ▸)
9	Allo-Si has a structure suggested similar to *allo*-Ge (similarity of powder XRD pattern), but never confirmed by Rietveld or single crystal data. A potential candidate for photovoltaic Si	Zeilinger *et al.* (2014 ▸)
10	CVD (chemical vapor deposition)	Hauge *et al.* (2015 ▸)
11	Hypothetical superstructures, a family of metastable BC8 structures	Rapp *et al.* (2015 ▸), Dmitrienko & Chizhikov (2020 ▸)
13 / 12 / 2	HPHT decomposition of some compounds can lead to Si-II crystallization and formation of Si-III in quasi-hydro­static conditions	Kurakevych *et al.* (2016 ▸)
14	Zeolite channel-like structure of Si_24_ directly transform to Si-4H polytype of diamond structure at slight heating	Shiell *et al.* (2021 ▸)
15	Hexagonal Si obtained by HPHT conditions is a 4H-polytype of diamond structure, as revealed by Rietveld refinement of XRD and TEM/SAED studies. Similar powder XRD patterns were reported in the paper	Pandolfi *et al.* (2018 ▸), Demishev *et al.* (1996 ▸)

## Data Availability

The data that support the findings of this study are available online and from the corresponding author upon reasonable request.
