# Supplementary material for: Silicon allotropes by large-volume high-pressure techniques: crystal growth mechanisms, phase diagrams and hexagonal nanostructured Si-6H by in situ X-ray diffraction and computational methods
Source: Acta Crystallogr B Struct Sci Cryst Eng Mater. 2026 Jun 1;82(Pt 3):280–98. doi: 10.1107/S2052520626004026 (PMC13238484; doi:10.1107/S2052520626004026)
Supplement: Supplementary file 2 [file b-82-00280-sup2.pdf]

STRUCTURAL SCIENCE
CRYSTAL ENGINEERING
MATERIALS

Volume 82 (2026)

Supporting information for article:

Silicon allotropes by large-volume high-pressure techniques: crystal growth mechanisms, phase diagrams and hexagonal nanostructured Si-6H by *in situ* XRD and computational methods

Alexandre Courac and Yann Le Godec

S1. In situ powder XRD of hexagonal Si phases at high temperature.

Rietveld refinement of Si-6H polytype is of better quality than that of Si-4H, due to the higher temperature of crystallization and reduction of stacking faults that increase the discrepancy between experimental and calculated profiles. Figure S1 show the refinement of the data with higher available to us wavelength (CuKα) that allow the best resolution of *hkl* reflexions. The stacking fault model of Warren type was used and allow significant improvement of fitting quality for Si-6H as compared to Si-4H.

Table S1 Fitting quality of Si-4H vs Si-6H.

The Rietveld refinement parameters extracted using MAUD software.

**Parameter**	**Si-4H (300 K)**	**Si-6H (873 K)**	**Remarks**
R_factor_all	0.0966	0.0493	
wR_factor_all	0.0738	0.0616	
goodness_of_fit_all	0.0496	0.0454	
cell_length_a	3.787 (0.003)	3.812 (0.001)	
cell_length_c	12.65 (0.02)	18.793 (0.009)	
cryst_size	99.5 (3.0)	Inf.	'Isotropic' size/strain
rs_microstrain	0.0078 (4E-4)	0.00797 (8E-5)	'Isotropic' size/strain
deformation_fault_intrinsic	0.05(0.004)	0.087 (0.010)	'Warren' planar defects
deformation_fault_extrinsic	0	0	'Warren' planar defects
twin_fault_probability	9.1E-7(5E-4)	0.22 (0.03)	'Warren' planar defects

Figure S1 Rietveld refinement of powder XRD of (a) Si-4H and (b) Si-6H at HT *in situ*.

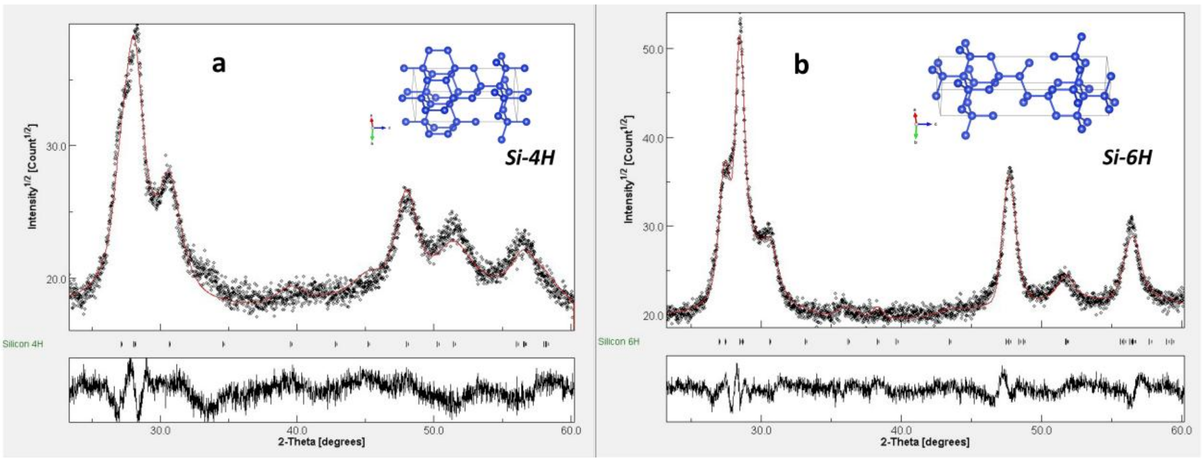
a
Si-4H
Intensity^1/2 [Count^1/2]
30.0
20.0
Silicon 4H
30.0
40.0
50.0
60.0
2-Theta [degrees]
b
Si-6H
50.0
40.0
Silicon 6H

Indexing of powder XRD of Si-VIII.

Identification of a new crystal structure by simply powder XRD can suffer from the possible contamination of materials with capsule, pressure gauge and/or pressure medium. The reported powder XRD in DAC (Zhao *et al.*, 1986) can be explained by a mixture of Si-III, Si-XII and Al_2O_3, well as expected (Figure S2).

Figure S2 Original powder XRD of presumed Si-VIII and simulated patterns of Si-III, Si-XII and Al_2O_3 mixture.

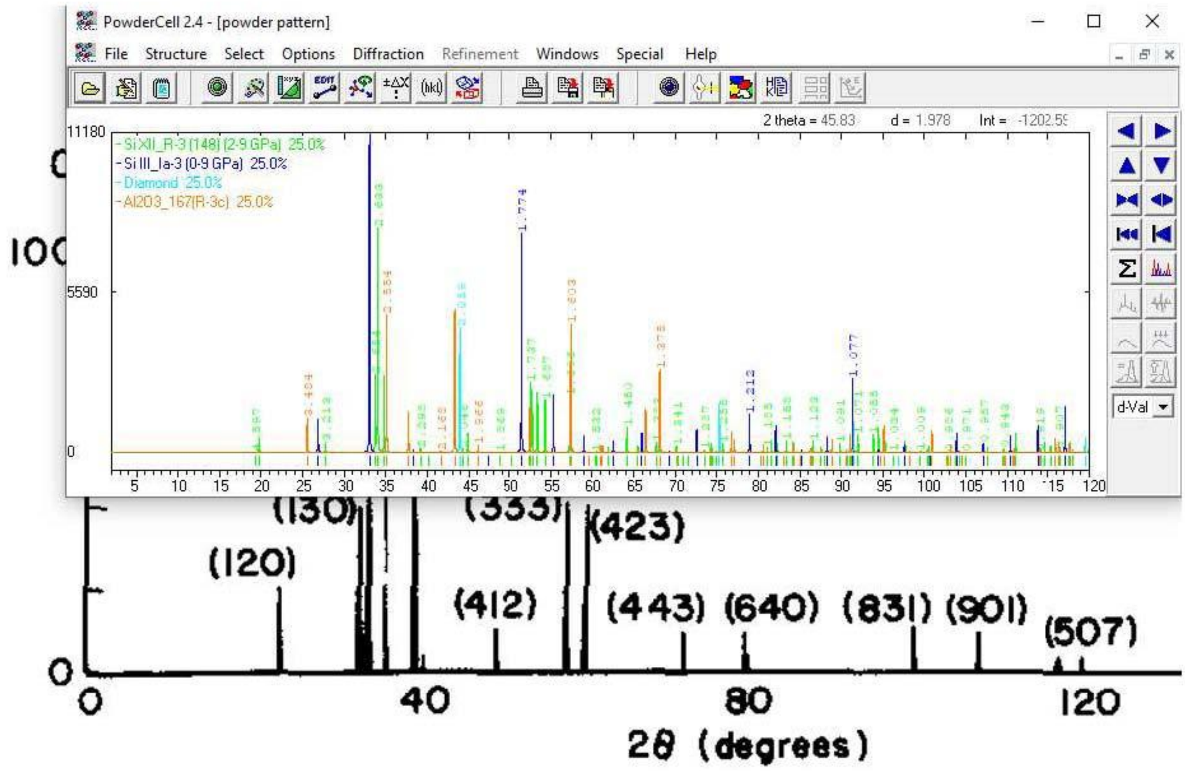
PowderCell 2.4 - [powder pattern]
File Structure Select Options Diffraction Refinement Windows Special Help
2 theta = 45.83 d = 1.978 Int = -1202.59
Si XII_R-3 (148) (2-9 GPa) 25.0%
Si III_Ia-3 (0-9 GPa) 25.0%
Diamond 25.0%
Al2O3_167(R-3c) 25.0%
11180
5590
0
d-Val
(130)
(333)
(423)
(120)
(412)
(443) (640) (831) (901)
(507)
0
40
80
120
2θ (degrees)

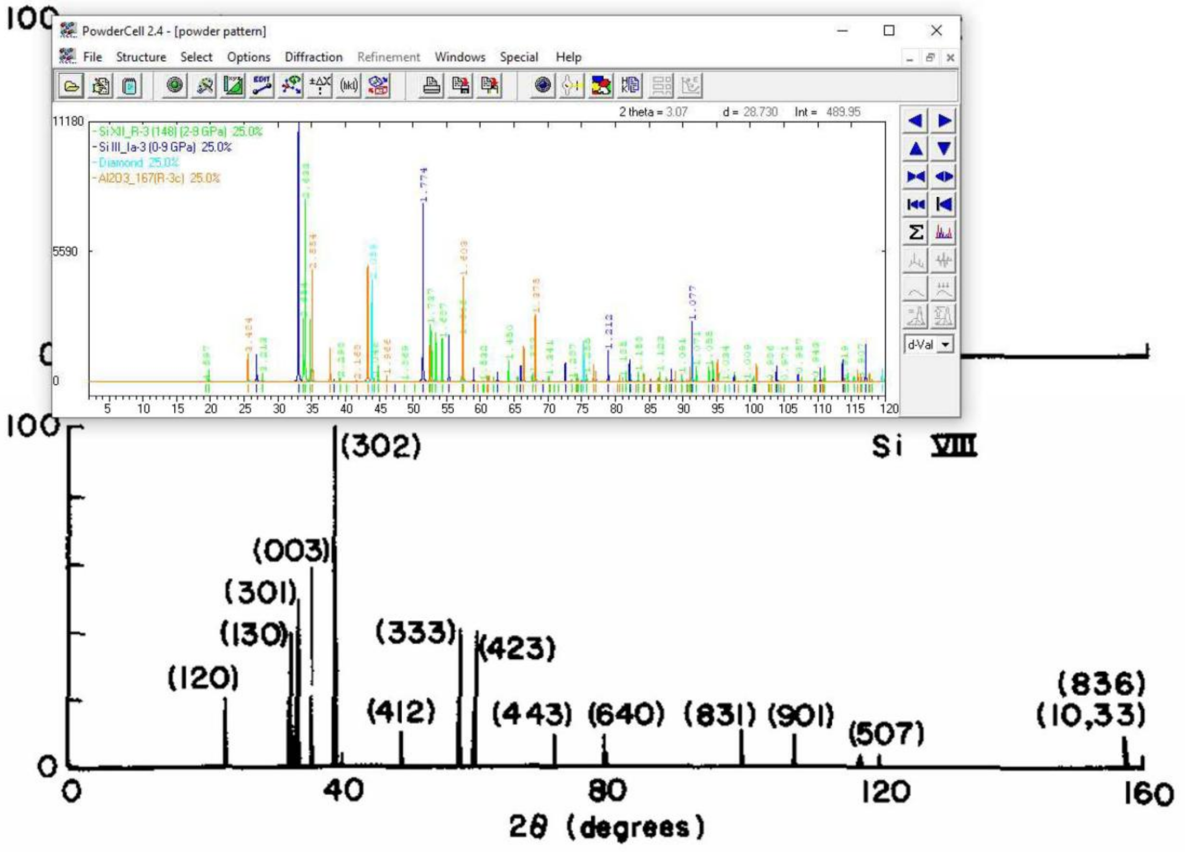
PowderCell 2.4 - [powder pattern]
File Structure Select Options Diffraction Refinement Windows Special Help
2 theta = 3.07 d = 28.730 Int = 489.95
Si XII_R-3 (148) (2-9 GPa) 25.0%
Si III_Ia-3 (0-9 GPa) 25.0%
Diamond 25.0%
Al2O3_167(R-3c) 25.0%
11180
5590
0
d-Val
100
(302)
Si VIII
(003)
(301)
(130)
(333)
(423)
(120)
(412)
(443) (640) (831) (901)
(507)
(836)
(10,33)
0
40
80
120
160
2θ (degrees)

Indexing of powder XRD of tetragonal Si-IX.

The reported powder XRD in DAC, suggested as a new single phase (Zhao *et al.*, 1986) can be satisfactorily explained by a mixture of Si-III, Si-XII and Al_2O_3, well as expected (Figure S3).

Figure S3 Original powder XRD of presumed Si-IX and simulated patterns of Si-III, Si-XII and Al_2O_3 mixture.

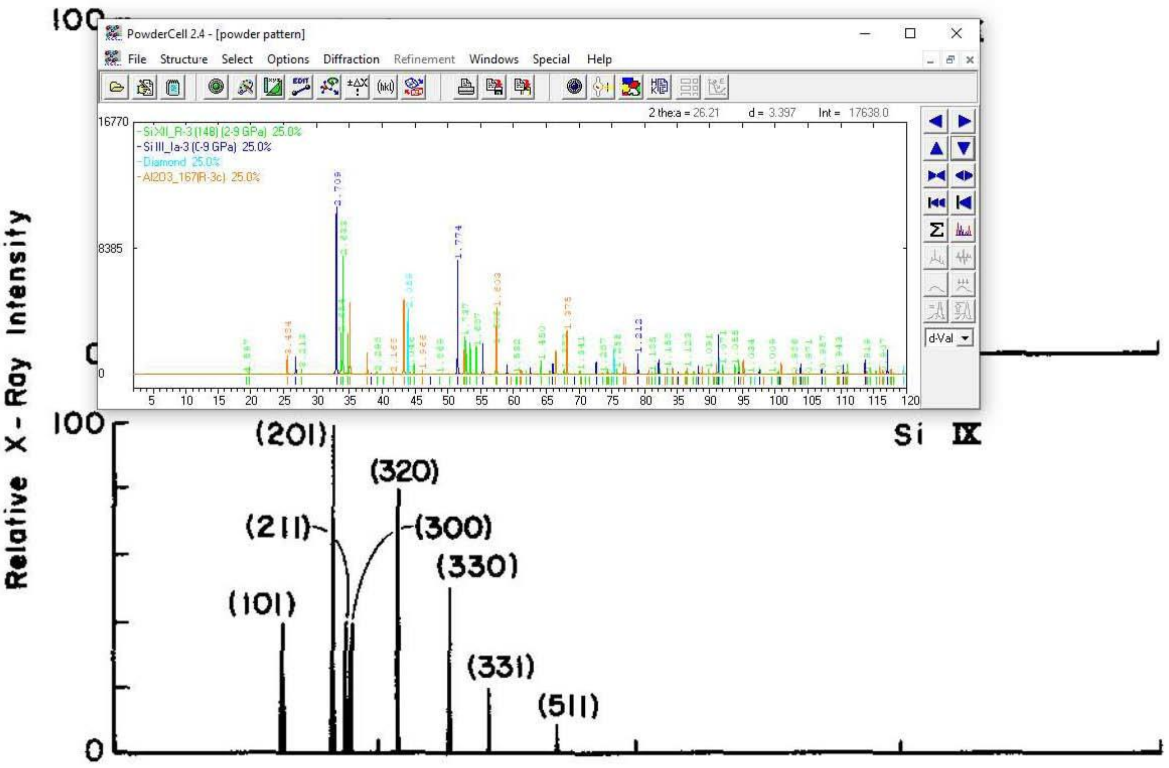

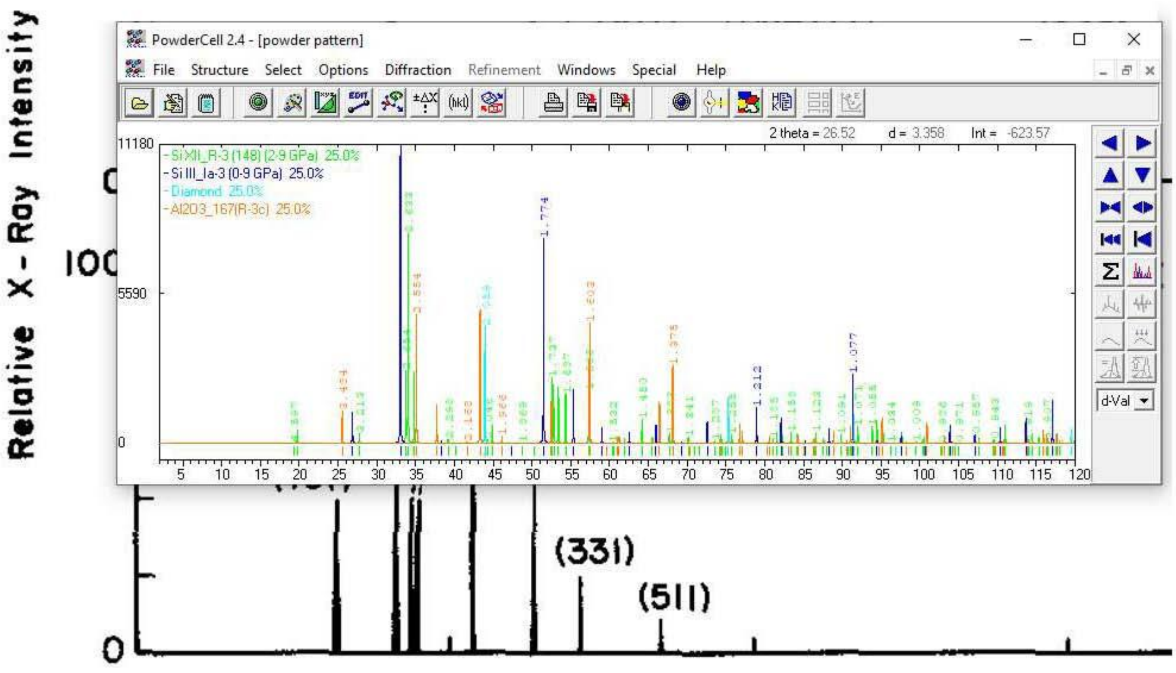
Relative X-Ray Intensity
PowderCell 2.4 - [powder pattern]
File Structure Select Options Diffraction Refinement Windows Special Help
2 theta = 26.52 d = 3.358 Int = -623.57
Si XII_R-3 (148) (2-9 GPa) 25.0%
Si III_Ia-3 (0-9 GPa) 25.0%
Diamond 25.0%
Al2O3_167(R-3c) 25.0%
11180
5590
0
d-Val
(331)
(511)
